# Root Traits Predict the Soil Functional Responses of Subtropical Plant Species to Experimental Drought

**DOI:** 10.1002/ece3.73048

**Published:** 2026-02-03

**Authors:** Lei Wang, Zewei Zhuang, Zheyang Su, Wenbin Li, Qizhi Wang, Xinfeng Chen, Ruiling Liu, Haoliang Lu, Yuxin Chen

**Affiliations:** ^1^ Key Laboratory of the Ministry of Education for Coastal and Wetland Ecosystems, College of the Environment & Ecology Xiamen University Xiamen China; ^2^ State Key Lab of Biocontrol and Guangdong Provincial Key Laboratory of Plant Stress Biology, School of Life Sciences Sun Yat‐Sen University Guangzhou China; ^3^ Guangzhou Field Station for Scientific Observation and Research of Urban Ecosystem Guangzhou Institute of Forestry and Landscape Architecture Guangzhou China; ^4^ Zhejiang Tiantong Forest Ecosystem National Observation and Research Station, Institute of Eco‐Chongming & Research Center for Global Change and Complex Ecosystems, School of Ecological and Environmental Sciences East China Normal University Shanghai China

**Keywords:** drought, habitat, plant trait, recovery, resistance, soil function

## Abstract

Substantial interspecific variation in both drought responses and soil functioning among woody species poses significant challenges for predicting drought impacts on soil functioning in species‐rich tropical and subtropical forests. However, critical knowledge gaps remain regarding how soil functions respond to drought across different plant species. We conducted a three‐phase (10 months of well‐watered conditions, 1 month of drought treatment, and 2 months of rewetting) seedling experiment to assess how drought impacts on eight rhizosphere soil functions related to carbon, nitrogen, and phosphorus cycling vary across 10 woody species. We tested whether plant species' preferences to arid versus moist habitats and functional traits could predict variation in the resistance and recovery of soil functions to drought. We found that soil functions of species adapted to the arid habitat or those possessing stronger drought‐tolerant traits (e.g., lower leaf water potential at turgor loss point) showed comparable resistance to their counterparts. Species with lower root N:P ratios and root non‐structural carbon concentrations consistently recovered faster in all four measured soil enzyme activities. Our results demonstrate that root chemical traits, particularly root N:P ratios and root non‐structural carbon concentrations, strongly predict soil enzyme activity recovery from drought. These findings significantly improve our understanding and prediction of drought impacts on soil functioning in species‐rich forests.

## Graphical abstract

Plant species' habitat preference and drought tolerance traits were not the primary factors driving interspecific differences in soil functional responses to drought. Instead, fine root traits—particularly their chemical traits—played more critical roles in determining these variations.

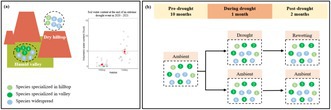

## 1 Introduction

Intensified droughts can severely impact forest ecosystem functioning by triggering widespread forest mortality and composition reorganization (Hartmann et al. 2018; Chen et al. 2025; Anderegg et al. 2020; Phillips et al. 2009). While numerous studies have documented drought effects on aboveground ecosystem functions, belowground processes (e.g., soil carbon and nutrient availability and soil enzyme activity) remain understudied (Mueller and Bahn 2022). Given the substantial interspecific variation in both drought responses (Williams and de Vries 2020) and linkages to soil functioning among plant species (de Vries et al. 2016; Phillips et al. 2013; Bardgett et al. 2014), predicting how drought alters soil functions in species‐rich tropical/subtropical forests presents a significant challenge. Addressing this challenge requires first understanding how different plant species vary in their responses to soil functions under drought.

The extracellular enzymes produced by rhizosphere microorganisms from different plant species play a critical role in regulating rhizosphere soil functions related to soil nutrient cycling and carbon (C) dynamics (Crowther et al. 2019; Kuzyakov and Razavi 2019). For example, β‐1,4‐*N*‐acetylglucosaminidase and acid phosphatase can cleave complex insoluble polymers (such as proteins, chitin, mononucleotides, phospholipids, and nucleic acids) into bioavailable nitrogen (N) and phosphorus (P) (Turner and Engelbrecht 2011; Schimel and Bennett 2004). Cellobiohydrolase and β‐1,4‐glucosidase can degrade cellulose (Han et al. 2023), increase labile organic carbon in soil, and affect C outputs from soil organic matter (SOM) mineralization (Canarini et al. 2017). Drought can impede plant carbon allocation to root and rhizosphere. The reduction of carbon allocation can reduce microbial extracellular enzyme activities (Hommel et al. 2016), and thus affect soil carbon and nutrient availabilities. Drought may also affect soil nutrient availabilities through changing plant uptakes of nutrients (de Vries et al. 2016). However, how extreme drought events affect rhizosphere soil enzyme activities, nutrients, and carbon cycling across different plant species in tropical/subtropical forests is still unclear.

Plant species' soil functional responses to drought likely correlate with their hydrological niche preferences. Even under identical rainfall regimes, pronounced topographic heterogeneity in subtropical mountainous ecosystems can generate substantial fine‐scale soil moisture variation, thereby driving spatially segregated plant community compositions (Peng et al. 2018). Plant species' habitat preferences emerge from long‐term eco‐evolutionary interactions with multiple environmental filters. For instance, drought‐adapted species typically exhibit aridity‐tolerant traits or enhanced soil nutrient cycling efficiency under water‐limited conditions (Geekiyanage et al. 2018; Li et al. 2012; Trugman et al. 2020). If drought‐induced changes in soil functions are critical for plant fitness during drought, a plant species' hydrological niche preference can well predict its soil functional responses to drought. However, whether plant species adapted to arid habitats are more resilient in soil functions to drought than species adapted to moist habitats remains poorly understood. Understanding interspecific variation in soil functional responses to drought across hydrological niche specialists is critical for predicting landscape‐scale drought impacts in topographically heterogeneous forests.

Traits governing plant water‐use strategies or drought tolerance may well predict species‐specific soil functional responses to drought if plant and soil systems exhibit coupled synchronized responses to water stress (Williams and de Vries 2020). However, this trait‐function relationship may be only evident during drought phases, whereas traits associated with resource acquisition strategies (e.g., root nitrogen concentration) likely better predict post‐drought recovery.

Different plant trait categories may vary in their capacities to forecast soil functional responses to drought. In contrast to leaves, roots can modify soil carbon and nutrient cycling directly by penetrating the soil, taking up water and nutrients, releasing root exudates, and through the process of root turnover (Bardgett et al. 2014). These properties of roots can make root traits (e.g., specific root length, root tissue density) likely outweigh leaf traits in predictive importance (Wan et al. 2021). Root chemical traits (e.g., root nitrogen concentration) are closely tied to microbial‐driven processes like decomposition and nutrient cycling. Therefore, root chemical traits may be more influential than root morphological traits in driving soil microbial activities (Wan et al. 2022; Han et al. 2023). The identities of functional traits that best predict soil functional responses to drought remain unresolved, limiting our ability to forecast forest ecosystem resilience to drought.

In this study, we aim to study how different subtropical woody species vary in their resistance and recovery of rhizosphere soil functions to drought. We conduct a seedling greenhouse experiment using 10 species from two habitats varying in soil water contents. We measure eight soil functions related to soil C, N, and P cycling after 1‐month drought and 2‐month rewetting, respectively. We hypothesize that: (1) Compared to species specialized to the moist habitat, species specialized to the arid habitat exhibit greater soil functional resistance to drought. (2) Plant species with stronger drought‐tolerant traits (e.g., lower leaf water potential at turgor loss point) exhibit higher resistance of soil functions to drought, but not necessarily faster recovery. (3) Different categories of plant traits differ in their predictive powers for species‐specific soil functional responses to drought. Specifically, traits with proximate soil linkages (root or chemical traits) outperform distal traits (leaf or morphological traits) in forecasting soil functional responses to drought.

## 2 Materials and Methods

### 2.1 Experimental Design

**FIGURE 1:**
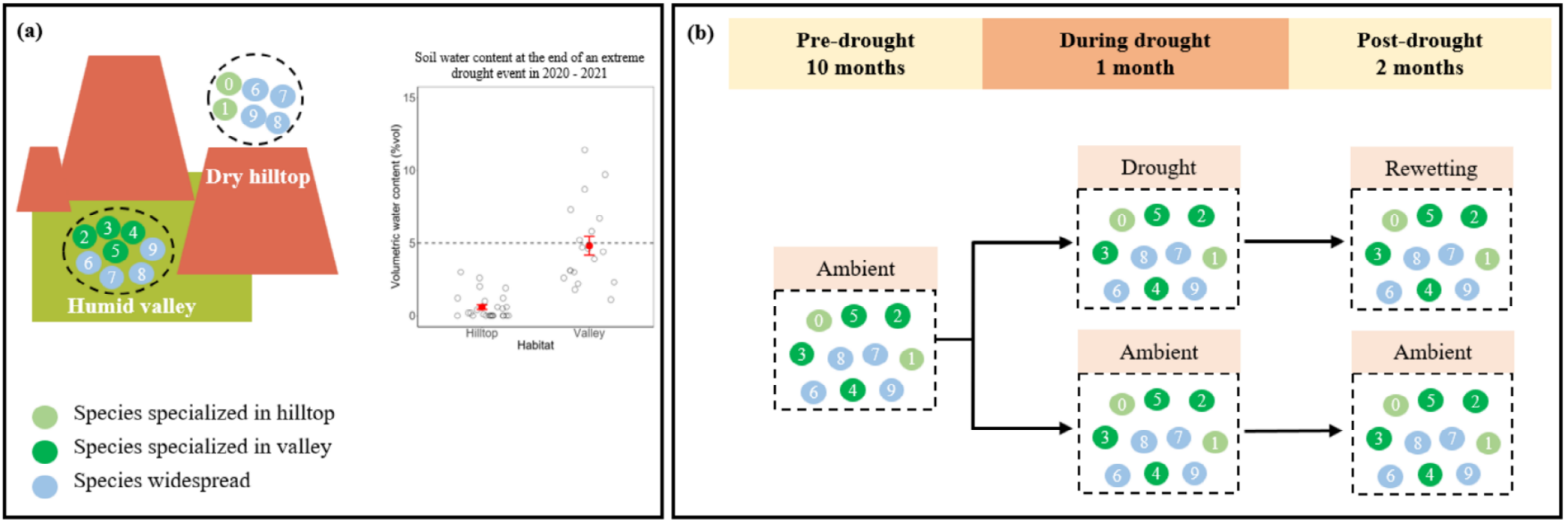
Experimental design. (a) The experiment used 10 species from the Danxia Landform that exhibit different habitat preferences between dry hilltop and humid valley. Circles in dark green, light green, and blue indicate species specialized in hilltop, specialized in valley, and widespread between habitats, respectively. The experiment simulated the extreme long drought in the field sites from late 2020 to early 2021. At the end of this drought event, the average soil volumetric water content (VWC) in the top 15 cm soil layers was below 5% for both habitats, as shown in the inset panel. Red filled points and error bars represent means ± standard error of soil VWC in each habitat, while empty circles represent raw data. (b) We conducted a three‐phase experiment involving ambient water (pre‐drought), drought stress (during drought), and rewetting (post‐drought) in a glasshouse. In the first phase, we grew 1‐year‐old seedlings under ambient soil moisture levels for 10 months. In the second phase, we treated half of the seedlings with drought stress and the other half with ambient water for 1 month. In the third phase, we rewetted the drought‐treated pots to ambient soil moisture levels as the ambient‐treated pots for 2 months.

To obtain woody species with diverse responses to drought, we collected seeds of 10 species from subtropical forests in a typical Danxia Landform, Taining World Natural Heritage Site in western Fujian Province, China (26°51′56″‐27°00′37″N, 117°02′22″‐117°13′07″E) in 2020 (Figure 1). The region has a subtropical monsoon humid climate, with mean annual precipitation of 1775 mm and mean annual temperature of 17.7°C. It experienced multiple years of extreme heat and drought events during 2019–2021 (Fujian Provincial Meteorological Bureau 2020–2022). In 2020, the region recorded an annual mean maximum temperature of 20.6°C, surpassing all previous records since 1961. Concurrently, a severe drought occurred, characterized by 50 consecutive days without effective precipitation. At the end of this drought event in the field, the average soil volumetric water content (VWC) was below 5% (Figure 1a).

Danxia Landform has distinctive geomorphology characterized by flat hilltops, steep cliffs and narrow valleys (Qi et al. 2005), which creates highly heterogeneous habitats and plant species compositions at fine spatial scales (Wu et al. 2008; Li et al. 2012; Peng et al. 2018). Compared to valleys, hilltops have thinner soil layers and lower soil water contents (Peng et al. 2018), likely supporting more drought‐adapted species. Based on the information of the species occurrence frequencies on hilltops and in valleys from local vegetation experts, we classified the 10 species into three categories: hilltop specialist with high frequency in hilltops but not in valleys, valley specialist with high frequency in valleys but not in hilltops, and generalist without apparent difference in distribution between the two habitats (Figure 1, Table 1).

**TABLE 1:** Summary information of woody species used in the experiment. Hilltop has lower soil water content than valley.

Species	Abbreviation	Family	Primary habitat	Leaf habitat
*Quercus phillyraeoides*	QUPH	Fagaceae	Hilltop	Evergreen
*Photinia serratifolia*	PHSE	Rosaceae	Hilltop	Evergreen
*Syzygium buxifolium*	SYBU	Myrtaceae	Generalist	Evergreen
*Lithocarpus glaber*	LIGL	Fagaceae	Generalist	Evergreen
*Ilex pubescens*	ILPU	Aquifoliaceae	Generalist	Evergreen
*Ormosia xylocarpa*	ORXY	Fabaceae	Valley	Evergreen
*Diospyros kaki* var.	DIKA	Ebenaceae	Generalist	Deciduous
*Rhus chinensis*	RHCH	Anacardiaceae	Valley	Deciduous
*Toxicodendron sylvestre*	TOSY	Anacardiaceae	Valley	Deciduous
*Camptotheca acuminata*	CAAC	Nyssaceae	Valley	Deciduous

To evaluate drought resistance and recovery of soil functions across species, we conducted a three‐phase experiment involving ambient water (pre‐drought), drought stress (during drought), and rewetting (post‐drought) in a glasshouse (Figure 1). In the first phase, we grew 1‐year‐old seedlings germinated from seeds under ambient soil moisture levels (20%–30% VWC) for 10 months. We planted 400 individuals (4 blocks × 10 species × 10 individuals) in September 2021. Each plant was transplanted into a pot (16 × 13 × 17.5 cm) with 2 kg sterilized soil collected on slopes between hilltops and valleys in Danxia Landform of Taining. Half of the seedlings received live rhizosphere soil (80 g per pot) of corresponding maternal trees in Taining, while the remaining half received the sterilized rhizosphere soil. The live soil was collected in a non‐drought, growing season of September 2021, 2 days before the transplantation. These soil sterilization and inoculation treatments were designed to preserve species‐specific soil microbial communities and assess plant–soil feedback in another study. In this study, we only used the seedlings inoculated with live rhizosphere soil. We replaced dead individuals within the first 2 weeks but not thereafter. In the second phase, we treated half of the seedlings with drought stress and the other half with ambient water for 1 month. For the drought‐stressed pots, we first simulated an extreme long drought without precipitation by stopping watering for 20 days, which reduced the soil moisture levels to 0%–5% VWC. We then simulated an extended drought period with minor precipitation by adding 15 mL water per pot per day in the remaining 2 weeks to keep the low soil moisture levels (0%–5% VWC). The experimental drought conditions were designed to simulate the extreme drought event that occurred in the Taining Danxia Landform during 2020–2021. At the end of this drought period, we conducted a field survey and found the average soil VWC at both hilltops and valleys was < 5% (Figure 1a). To monitor drought progression, we randomly selected 20 drought‐stressed pots for soil moisture measurement using the WET Sensor per day. In the third phase, we rewetted the drought‐treated pots to ambient soil moisture levels (20%–30% VWC), as the ambient‐treated pots for 2 months. We harvested one individual per species per treatment level per block at the end of the first and second phases, respectively, and all the remaining live plants at end of the experiment. The sample size and replication statement were available in Table S1. While most species maintained 3–4 replicates, species *Lithocarpus glaber* (LIGL) was underrepresented during recovery (0 in control, 3 in drought treatment). Consequently, this species was omitted from the recovery‐phase analysis. Within each block, the positions of pots subjected to different drought and species treatments were randomized biweekly.

### 2.2 Rhizosphere Soil Functions

To assess the effects of drought and rewetting on soil functions, we measured four rhizosphere soil enzyme activities and four rhizosphere soil carbon and nutrient availabilities. We collected one sample of rhizosphere soil per harvested plant. We gently shook root branches and collected the soil adhering to the root surface. The soil samples were sieved to 2 mm and stored at 4°C. We measured the soil enzyme activities of cellobiohydrolase (CBH, nmol MUF g^−1^ h^−1^), β‐1,4‐glucosidase (BG, nmol MUF g^−1^ h^−1^), β‐1,4‐*N*‐acetylglucosaminidase (NAG, nmol MUF g^−1^ h^−1^), and acid phosphatase (AP, nmol MUF g^−1^ h^−1^) using the 96‐well microplates according to Saiya‐Cork et al. (2002). In brief, fresh rhizosphere soil of 1 g was suspended in 125 mL of sodium acetate buffer. We adjusted the pH of the buffer to 5, which was close to the average pH of soil samples. We homogenized the soil and buffer using a magnetic stirring apparatus (3 min, 200 rpm). We combined 200 μL of soil slurry and the substrate solution (50 μL) in each well of the microplates. These microplates were then incubated in the dark at 25°C for 4 h. We used 4‐methylumbelliferyl‐β‐D‐cellobioside, 4‐methylumbelliferyl‐β‐D‐glucoside, 4‐methylumbelliferyl‐*N*‐acetyl‐β‐D‐glucosaminide, and 4‐methylumbelliferyl‐phosphate as substrates for CBH, BG, NAG, and AP, respectively. To stop the reaction, a 10 μL aliquot of 1 mol L^−1^ NaOH was added to each well. After incubation, we measured the amount of fluorescence for enzymes at 365 nm excitation and 450 nm emission.

For rhizosphere soil carbon and nutrient availabilities, we measured dissolved organic carbon (DOC), available phosphorus (${\mathrm{PO}}_{4}^{3-}$), ammonium nitrogen (${\mathrm{NH}}_{4}^{+}$), and nitrate nitrogen (${\mathrm{NO}}_{3}^{-}$). We followed the method of Curtin et al. (2006) to extract DOC from 10 g of fresh soil with 40 mL of deionized water. The filtrate was obtained through 0.45 μm membranes. Extractable organic carbon was quantified using a TOC analyzer (SHIMADZU TOC‐VCPH/CPN, Japan). ${\mathrm{PO}}_{4}^{3-}$ was extracted from 5 g of dry soil with 40 mL of ammonium fluoride‐hydrochloric acid solution. ${\mathrm{NH}}_{4}^{+}$ and ${\mathrm{NO}}_{3}^{-}$ were extracted from 40 g of fresh soil with 200 mL of potassium chloride solution. The concentrations of ${\mathrm{PO}}_{4}^{3-}$, ${\mathrm{NH}}_{4}^{+}$, and ${\mathrm{NO}}_{3}^{-}$ were determined by spectrophotometric methods.

### 2.3 Plant Functional Traits

To evaluate why species varied in their responses of rhizosphere soil functions to drought, we measured 14 chemical or morphological traits from fine roots or leaves, which are closely associated with plant tolerance to drought or resource usage strategies (Table 2) (Han et al. 2023; Wan et al. 2022; Freschet et al. 2021; Alvarez‐Cansino et al. 2022). All these traits were measured from plant without drought stress. For each species, we selected one individual per block to measure all traits (*n* ≥ 3), except for specific leaf area (SLA), which was sampled more intensively (*n* ≥ 4) (Table S2). We averaged these raw trait values to obtain species‐level means.

**TABLE 2:** Summary information of root and leaf traits.

Plant trait	Abbreviation	Unit
Root nitrogen concentration	RN	g kg^−1^
Root phosphorus concentration	RP	g kg^−1^
Root nitrogen: phosphorus ratio	RNP	—
Root non‐structural carbohydrate concentration	RNSC	mg g^−1^
Root diameter	RD	mm
Root branching intensity	RBI	tips cm^−1^
Specific root area	SRA	cm^2^ g^−1^
Proportion of mycorrhizal fungi among fungi	MF	%
Leaf water potential at turgor loss point	TLP	MPa
Leaf nitrogen concentration	LN	g kg^−1^
Leaf nitrogen: phosphorus ratio	LNP	—
Leaf non‐structural carbohydrate concentration	LNSC	mg g^−1^
Leaf area	LA	mm^2^
Specific leaf area	SLA	mm^2^ g^−1^

*Note:* “—” denotes no unit.

For each plant, one mature and intact leaf was collected to measure leaf water potential at turgor loss point (TLP). We used rehydrated leaves to measure TLP by the squeeze method (Lawren et al. 2023; Bartlett et al. 2012). TLP is a good predictor for drought resistance of woody species (Alvarez‐Cansino et al. 2022). We sealed each leaf in a self‐sealing bag filled with water and wrapped in triple‐layer black polyethylene bags. We refrigerated the leaves overnight and measured leaf water potential in the following day using a portable pressure chamber (Model 1505D‐EXP; PMS Instrument Company, Albany, USA). Leaf water potential was measured at different water loss gradients to construct pressure‐volume curves. Under ventilated conditions, we adjusted the measurement intervals according to the water loss rate of different species, ranging from 10 to 30 min. We continued the measurement until either seven gradients were recorded or the leaf water potential reached −3.0 MPa. TLP was determined as the inflection point where pressure‐volume curves transitioned from curvilinear to linear segments.

After the measurement of TLP, we dried the target leaf samples at 65°C for 72 h to determine dry mass. SLA was calculated by dividing leaf area (LA) by leaf dry mass.

To measure leaf non‐structural carbohydrate concentration (LNSC, including starch and soluble sugar concentrations), we dried the leaves at 110°C for 15 min to stop enzymatic activity immediately after collection. These samples were then oven‐dried at 65°C and ground into fine powders using an automated ball mill. LNSC was analyzed as described by Zhang et al. (2024) with minor modifications. Briefly, 50 mg of the leaf fine powders were extracted with 4 mL of 80% (v/v) ethanol and then incubated in a water bath at 100°C for 10 min. The supernatants obtained by twice centrifuging were combined and used for soluble sugar determination. Starch was released from the ethanol‐insoluble residual separated after extraction by boiling in distilled water and was then successively digested with α‐amylase and amyloglucosidase. The supernatants gained after centrifugation were used for starch determination. The concentrations of soluble sugars and starch were measured using the anthrone colorimetric method with a UV–visible spectrophotometer at a wavelength of 625 nm (Dubois et al. 1951). Starch concentration was calculated by multiplying a reference concentration of glucose by the conversion factor of 0.9.

Leaf nitrogen concentration (LN) was quantified via sulfuric acid‐hydrogen peroxide digestion with automated Kjeldahl analysis. Leaf phosphorus concentration (LP) was quantified via sulfuric acid‐hydrogen peroxide digestion with determination by spectrophotometers.

We collected three fine root branch samples (< 2 mm diameter) per plant to measure root traits. Root morphological traits included root diameter (RD), specific root area (SRA), and root branching intensity (RBI). We scanned the cleaned root branch using an Epson Perfection V850 Pro scanner. These images were analyzed with WinRHIZO Pro software to determine the mean RD, total length, total surface area, and root tip number. Then all root branches were dried at 65°C for 72 h to a constant mass and weighed. RBI was calculated from the tip number divided by the total root length. SRA was calculated as total surface area divided by root dry mass.

Root chemical traits were determined using the same methods as foliar analyses. These include root nitrogen concentration (RN), root phosphorus concentration (RP), root N:P ratio (RNP), and root non‐structural carbohydrates concentration (RNSC).

We measured mycorrhizal fungal proportion (MF) within roots as the relative abundance of arbuscular mycorrhizal fungi and ectomycorrhizal fungi to all fungi. We identified fungal identities and relative abundances through amplicon sequencing of root endosphere fungi. For root endosphere fungi, the ITS1‐1F region was amplified using the universal primers ITS1F (CTTGGTCATTTAGAGGAAGTAA) and ITS2 (GCTGCGTTCTTCATCGATGC) (Johnston‐Monje et al. 2021). The library was sequenced on an Illumina NovaSeq platform. Detailed information about the sequencing and bioinformatic analyses can be found in the Supporting Information.

We excluded plant traits exhibiting absolute correlations > 0.7 within leaf traits or within fine root traits. Leaf C: N ratio, leaf C:P ratio, leaf dry matter content, leaf phosphorus concentration, root C: N ratio, root C:P ratio, specific root length, root dry matter content, and root tissue density were excluded. After the exclusion, we retained 14 traits for subsequent analyses (Figure S1).

We also tried to assess whether differences in drought responses in plant relative growth rates (RGR) among species were associated with their soil functional responses. However, we did not find significant species variation in their drought resistance or recovery of RGR. Species' average RGR under ambient conditions was not significantly correlated with soil functional responses to drought. These preliminary analysis results led us to focus on the linkages between the above‐mentioned species traits from ambient conditions and soil functional responses to drought.

### 2.4 Statistical Analyses

We performed general linear models (model 1) to assess the effects of drought and rewetting treatments on soil functions by setting block, species, treatment, and species × treatment as explanatory variables in this order. We tested the significance of treatment across species by using its interaction with species as an error term. This procedure treated the species as the fundamental unit of replication (Schmid et al. 2017; Chen et al. 2022). In preliminary analysis, we also tried linear mixed‐effects models, in which we set block and drought treatment as fixed‐effect terms, species and its interaction with treatment as random‐effect terms. We fitted these mixed‐effects models using the lmer function with default settings in the lme4 package (Bates et al. 2015). However, the vast majority of these models encountered singular fit or boundary issues. Therefore, we only reported the results from general linear models. We tested the significance of treatment per species based on these general linear models using the emmeans R package (Lenth 2023). We square‐root transformed CBH and log‐transformed BG, AP, NAG, DOC, ${\mathrm{PO}}_{4}^{3-}$, ${\mathrm{NH}}_{4}^{+}$, and ${\mathrm{NO}}_{3}^{-}$ before fitting the models to improve residual normality. We repeated the above analyses separately for each soil function. We did not account for multiple testing because we aimed to identify which traits best predict the response of specific soil functions, but not to generalize a trait's importance for all functions based on a single significant relationship.

To assess the differences in soil functions of drought‐treated plants between drought and rewetting phases, we performed general linear models similar to model 1, except we replaced the term treatment with phase.

To examine differences in the effects of drought and rewetting on soil function between species groups (e.g., habitat generalist vs. specialist, hilltop specialist vs. valley specialist), we performed general linear models by setting block, species group, species, treatment, species group × treatment, and species × treatment as explanatory variables in this order. We tested the significance of species group × treatment by using species × treatment as an error term. We calculated the mean treatment effects of each species group using the emmeans R package (Lenth 2023). We also compared differences in the effects of drought and subsequent rewetting on soil function between evergreen and deciduous species but did not find significant differences.

We performed weighted linear regression to assess the effects of plant traits on species‐specific functional responses to drought and rewetting. In these models, we set species‐specific average response to drought or rewetting as the response variable, the inverse of its standard error as weight, and each trait as explanatory variables. We conducted the analysis for each individual trait separately, due to the high correlation between some of these traits (Figure S1). These models considered the species as the fundamental unit of replication. Each trait was standardized (mean zero and unit standard deviation) before regression. Therefore, the standardized effect sizes not only allowed us to assess which traits were more important but also revealed whether each trait had a positive or negative effect on the soil functional response to drought. We calculated species‐specific effect of drought and rewetting on soil function from model 1. In preliminary analysis, we tried linear mixed‐effects models using all raw observations (i.e., difference in soil functions between drought and ambient treatments per species per block) as the response variable, with species included as a random intercept, and block and trait as fixed effects. However, the vast majority of these models encountered singular fit or boundary issues. Therefore, we only reported the results from weighted regressions. A sensitivity analysis, in which we excluded the most influential outlier (Cook's distance > 1) from the regression between soil functional resistance and recovery and plant traits, yielded conclusions similar to those from the full dataset (Figures S2 and S3).

To assess the effects of multiple traits on species‐specific functional responses to drought, we repeated the above analyses by substituting individual traits with principal component analyses (PCA) scores of multiple traits. We used species‐level traits to conduct PCA using the FactoMineR package (Lê et al. 2008). We obtained the first (PC1) and second (PC2) axes scores from the PCA for the regressions. These two axes accounted for 52.9% of the variance of the total variation in traits (Figure S4). We log‐transformed LN, LNP, RD, RBI, MF, RNSC, RN, RP, RNP, and the absolute value of TLP before the PCA to improve normality.

All statistical analyses were conducted using R v.4.4.3 (R Core Team 2025). We presented the figures using the ggplot2 R package (Wickham 2016). Data and R scripts for this study are available at figshare via the identifier (https://figshare.com/s/90b66f9ca86690316aa1).

## 3 Results

### 3.1 Effects of Drought and Rewetting on Rhizosphere Soil Functions

**FIGURE 2:**
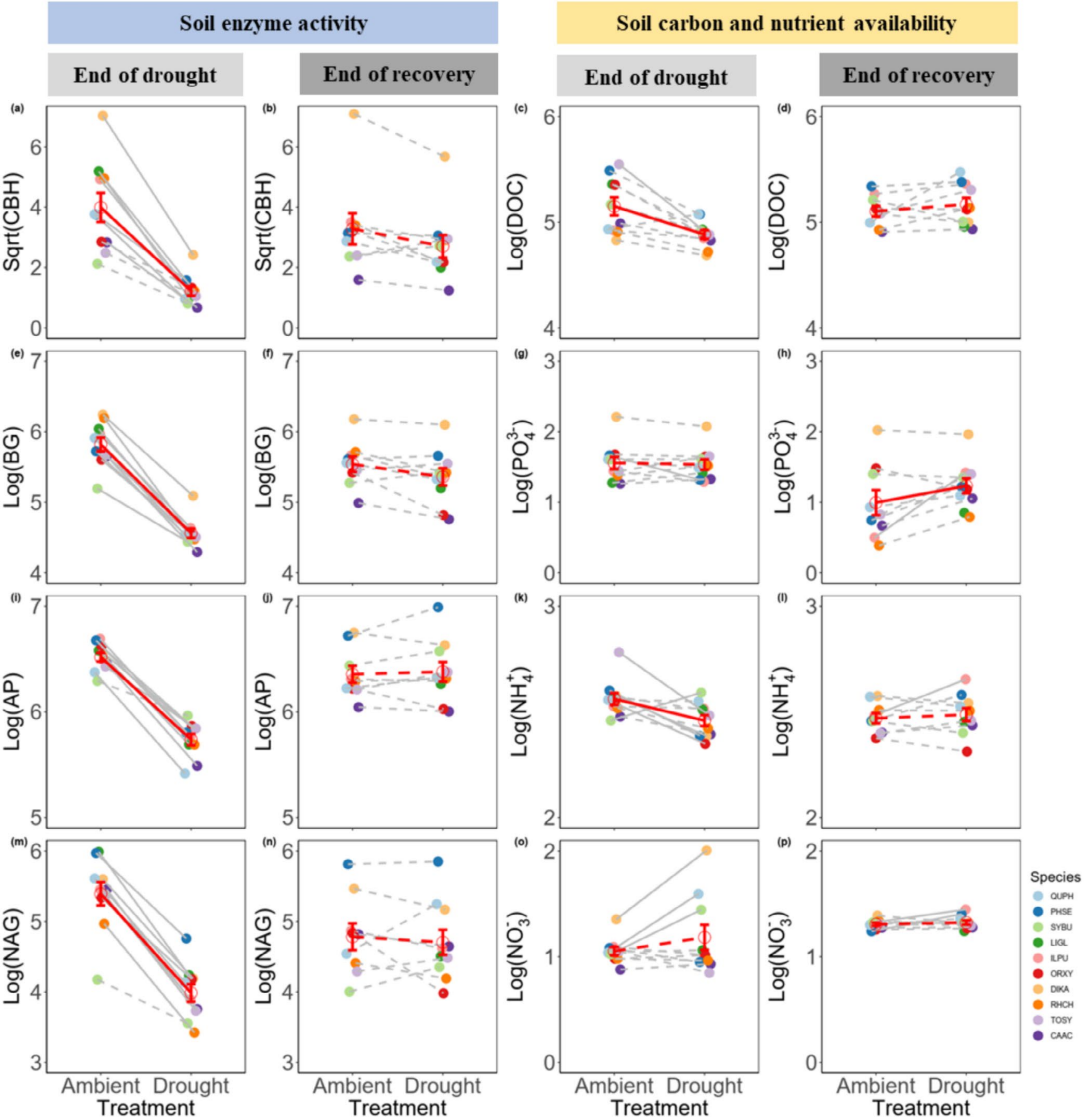
Effects of drought (end of drought) and rewetting after drought (end of recovery) on rhizosphere soil enzyme activity, and carbon and nutrient availability. Soil enzyme activities include cellobiohydrolase (CBH, nmol MUF g^−1^ h^−1^, a and b), β‐1,4‐glucosidase (BG, nmol MUF g^−1^ h^−1^, e and f), acid phosphatase (AP, nmol MUF g^−1^ h^−1^, i and j), β‐1,4‐*N*‐acetylglucosaminidase (NAG, nmol MUF g^−1^ h^−1^, m and n). Soil carbon and nutrient availabilities include dissolved organic carbon (DOC, mg kg^−1^, c and d), available phosphorus (${\mathrm{PO}}_{4}^{3-}$, mg kg^−1^, g and h), ammonium nitrogen (${\mathrm{NH}}_{4}^{+}$, mg kg^−1^, k and l), and nitrate nitrogen (${\mathrm{NO}}_{3}^{-}$, mg kg^−1^, o and p). Red empty points and error bars represent means ± standard error of soil variables across different tree species for the corresponding treatment. Red solid lines indicate significant differences (*p* < 0.05) in the mean values across species between drought and ambient treatments, while red dashed lines denote non‐significant differences (*p* > 0.05, Tables S3 and S4). Solid circles indicate species‐specific means. Gray solid lines indicate significant differences (*p* < 0.05) between drought and ambient treatments for individual species, while gray dashed lines indicate non‐significant results (*p* > 0.05, Tables S5–S8). Full species names are listed in Table 1. Data for ambient treatment of tree species *Lithocarpus glaber* (LIGL) during recovery phase are missing due to insufficient plant samples.

The extreme drought significantly reduced all four rhizosphere soil enzyme activities (CBH, BG, AP, NAG) for most species (Figure 2, Tables S3 and S5). However, most species showed weak responses of the soil carbon and nutrient availabilities to drought (Figure 2, Tables S3 and S6). When averaging across species, drought significantly reduced DOC and ${\mathrm{NH}}_{4}^{+}$, but not ${\mathrm{PO}}_{4}^{3-}$ and ${\mathrm{NO}}_{3}^{-}$ (Figure 2, Tables S3 and S6). The four soil enzyme activities and DOC quickly recovered after drought (Figure S5 and Table S9) and returned to ambient levels after two months rewetting (Figure 2, Tables S4, S7 and S8). However, ${\mathrm{NH}}_{4}^{+}$ did not show significant recovery after drought (Figure S5), despite similar drought impacts and similar values at the end of recovery between rewetting and ambient plants (Figure 2). Surprisingly, ${\mathrm{PO}}_{4}^{3-}$ of drought‐stressed plant species decreased after rewetting (Figure S5), but increased relative to that of plant species under ambient conditions at the end of recovery (Figure 2).

We did not find consistent differences in soil functional responses to drought among plant species with different habitat preferences. Specifically, the resistances and recoveries of soil functions did not differ significantly between species widespread and generalized to specific habitats (Figures S6 and S7, Table S10). Species specialized in hilltop and valley did not differ significantly in their resistances nor recoveries of soil functions, with the exception of the resistance of ${\mathrm{PO}}_{4}^{3-}$ and the exception of the recovery of ${\mathrm{NO}}_{3}^{-}$ (Figures S8 and S9, Table S11).

### 3.2 Effects of Plant Trait on Species‐Specific Response of Rhizosphere Soil Function to Drought and Rewetting

**FIGURE 3:**
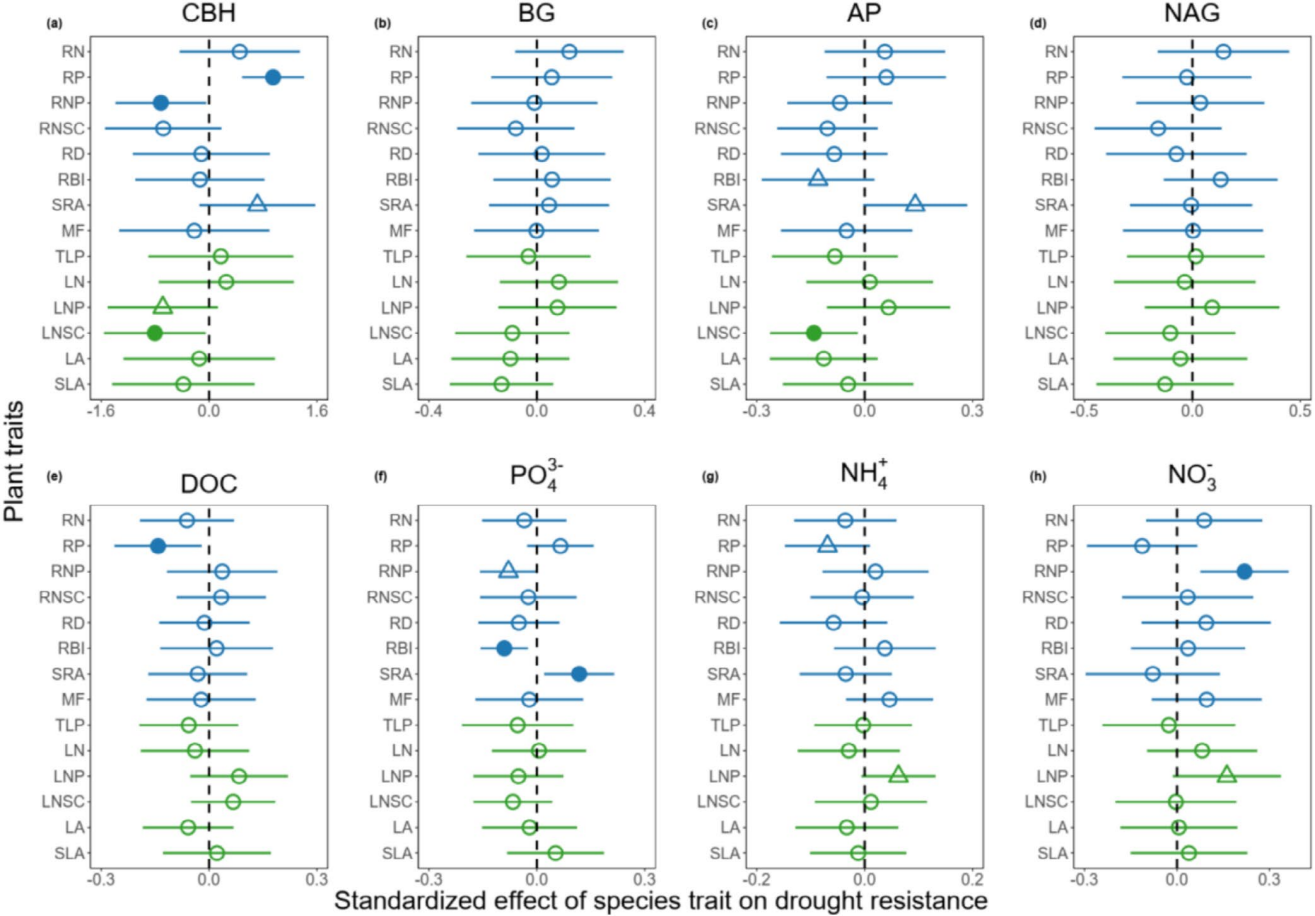
Effects of plant functional traits on species‐specific resistance of rhizosphere soil enzyme activity (a–d), carbon and nutrient availability (e–h) to drought. Soil enzyme activities include cellobiohydrolase (CBH), β‐1,4‐glucosidase (BG), acid phosphatase (AP), and β‐1,4‐*N*‐acetylglucosaminidase (NAG). Soil carbon and nutrient availabilities include dissolved organic carbon (DOC), available phosphorus (${\mathrm{PO}}_{4}^{3-}$), ammonium nitrogen (${\mathrm{NH}}_{4}^{+}$), and nitrate nitrogen (${\mathrm{NO}}_{3}^{-}$). Points and lines represent mean standardized effects and their 95% confidence intervals (CIs), respectively. Blue and green symbols indicate results from fine root and leaf traits. Solid circles indicate statistically significant effects if the 95% CIs exclude zero; triangles indicate marginally significant effects if the 90% CIs exclude zero; empty circles indicate non‐significant effects if the 90% CIs include zero. The full name of plant traits can be found in Table 2.

Four root and leaf chemical traits (RP, RNP, LNP, and LNSC) were among the best predictors of species‐specific resistances of soil functions to drought, although the most important traits may vary for different soil functions (Figure 3). For CBH, species with higher RP, lower RNP, lower LNP, or lower LNSC showed higher resistance; for ${\mathrm{NO}}_{3}^{-}$, species with higher RNP or LNP were more resistant to drought; for ${\mathrm{NH}}_{4}^{+}$, species with lower RP or higher LNP were more resistant to drought. Species with lower LNSC, RP, and RNP tended to show higher resistance in AP, DOC, and ${\mathrm{PO}}_{4}^{3-}$, respectively.

In addition to chemical traits, two root morphological traits (SRA and RBI) were also important in determining species resistance of soil functions to drought (Figure 3). Species with higher SRA showed higher resistance in CBH, AP, and ${\mathrm{PO}}_{4}^{3-}$; species with lower RBI showed higher resistance in AP and ${\mathrm{PO}}_{4}^{3-}$.

Inconsistent with our expectation, traits critical for plant tolerance to drought (TLP) were not significantly related to species' soil functional resistance (Figure 3). Species' positions along multi‐trait PCA axis 2 exhibited stronger influence on soil functional resistance than axis 1 (Figures S4 and S10), as this axis captured more variance in the critical traits governing soil functional resistance (Figures S4, S11 and S12).

**FIGURE 4:**
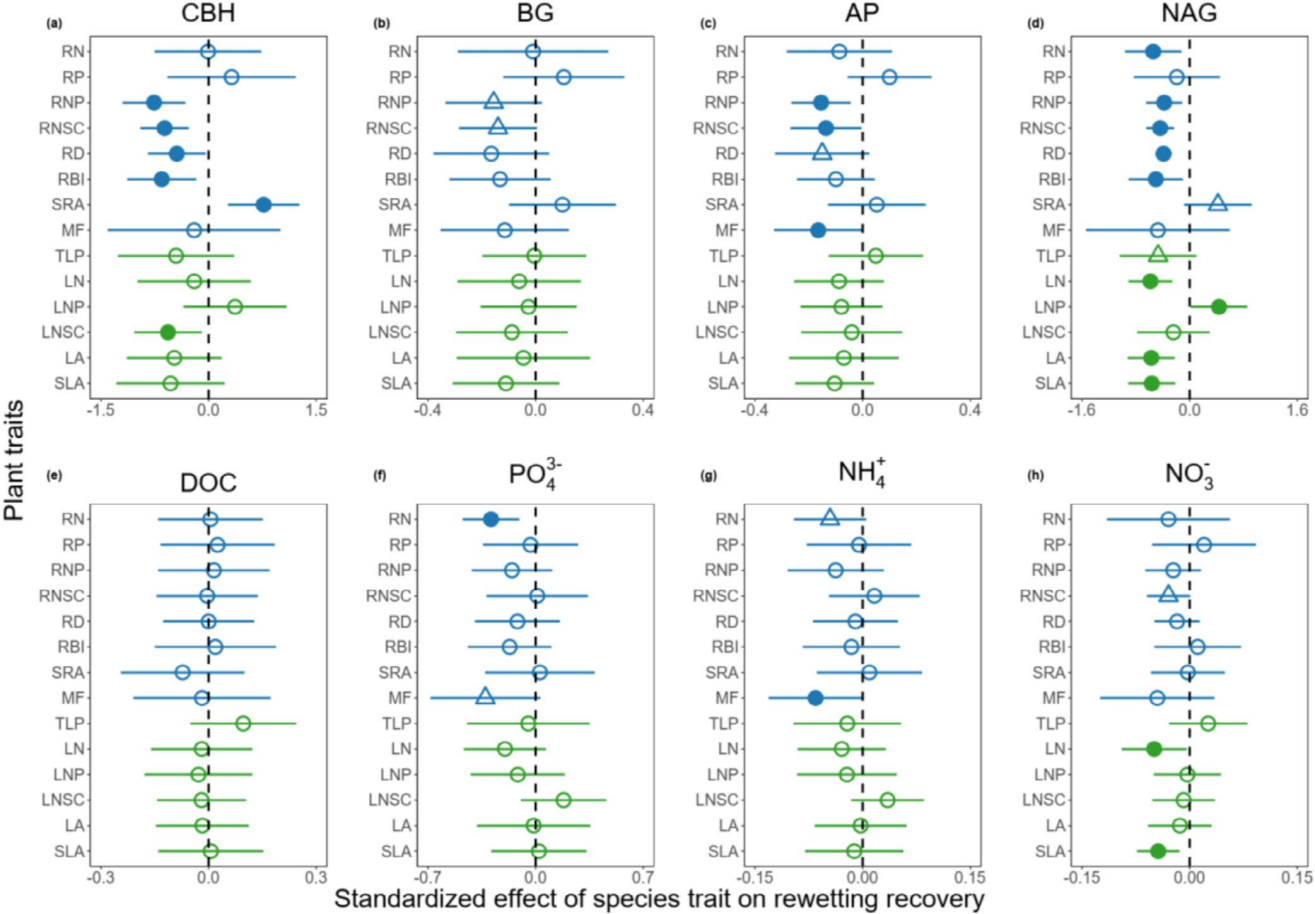
Effects of plant functional traits on species‐specific recovery of rhizosphere soil enzyme activity (a–d), carbon and nutrient availability (e–h) from rewetting. Soil enzyme activities include cellobiohydrolase (CBH), β‐1,4‐glucosidase (BG), acid phosphatase (AP), and β‐1,4‐*N*‐acetylglucosaminidase (NAG). Soil carbon and nutrient availabilities include dissolved organic carbon (DOC), available phosphorus (${\mathrm{PO}}_{4}^{3-}$), ammonium nitrogen (${\mathrm{NH}}_{4}^{+}$), and nitrate nitrogen (${\mathrm{NO}}_{3}^{-}$). Points and lines represent mean standardized effects and their 95% confidence intervals (CIs), respectively. Blue and green symbols indicate results from fine root and leaf traits. Solid circles indicate statistically significant effects if the 95% CIs exclude zero; triangles indicate marginally significant effects if the 90% CIs exclude zero; empty circles indicate non‐significant effects if the 90% CIs include zero. The full name of plant traits can be found in Table 2.

**FIGURE 5:**
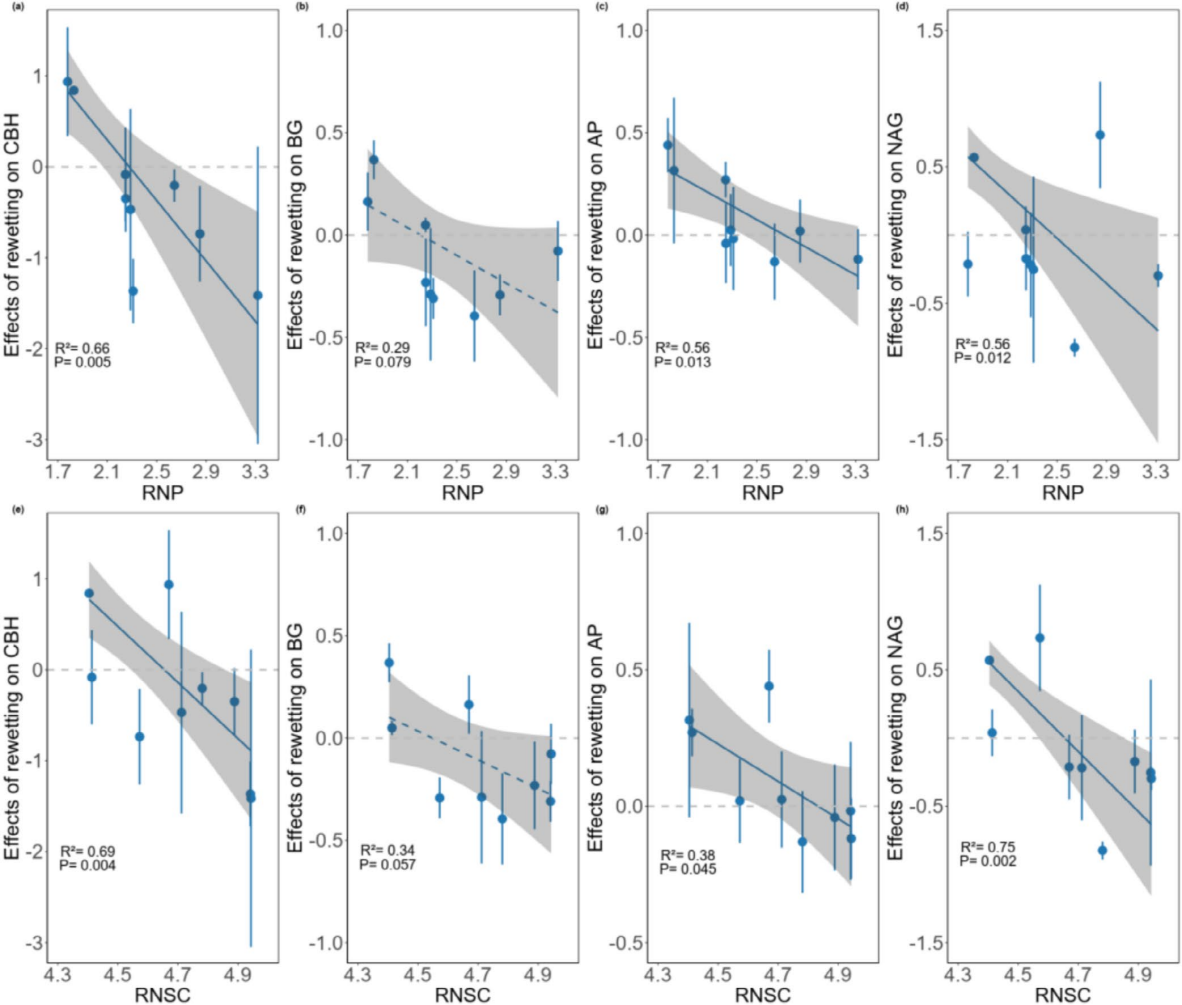
Rhizosphere soil enzyme activities recovered slower for species with higher root N:P ratios (RNP) and root non‐structural carbon concentrations (RNSC) after rewetting. Soil enzyme activities include cellobiohydrolase (CBH; a, e), β‐1,4‐glucosidase (BG; b, f), acid phosphatase (AP; c, g), and β‐1,4‐*N*‐ acetylglucosaminidase (NAG; d, h). Blue points and error bars display means ± standard errors of rewetting effects per species. Blue lines represent the predicted relationships between species‐specific effects of rewetting and root traits, with solid and dashed lines indicate significant (*p* < 0.05) and marginally significant (*p* < 0.1) results, respectively. Gray shaded areas indicate 95% CIs of predicted relationships. Data of tree species *Lithocarpus glaber* (LIGL) under ambient treatment during rewetting phase are missing due to insufficient plant samples.

Root traits were important in determining species‐specific recovery of soil enzyme activities. The four soil enzyme activities recovered more slowly for species with higher RNP or RNSC (Figures 4 and 5). A one‐SD increase in RNSC was associated with a decrease of 0.614 (95% CI: 0.276, 0.952), 0.140 (95% CI: 0.005, 0.285), 0.137 (95% CI: 0.004, 0.268), and 0.438 (95% CI: 0.232, 0.644) nmol g^−1^ h^−1^ in the recovery of CHB, BG, AP, and NAG, respectively; a one‐SD increase in RNP was associated with a decrease of 0.761 (95% CI: 0.322, 1.199), 0.156 (95% CI: −0.023, 0.335), 0.154 (95% CI: 0.044, 0.265), and 0.379 (95% CI: 0.110, 0.647) nmol MUF g^−1^ h^−1^ in the recovery of CHB, BG, AP, and NAG, respectively. Species with lower RD or RBI tended to recover faster in CBH, AP, and NAG; species with higher SRA tended to recover faster in CBH and NAG (Figure 4). As these root traits had significant contributions to the second axis of PCA of multiple traits (PC2, Figures S4, S11 and S12), PC2 had consistently important effects on the recovery of soil enzyme activities (Figure S13). In addition to root traits, leaf traits were also important in determining species recovery of NAG (Figure 4).

Compared with soil enzyme activities, functional traits have relatively poorer predictive performance and consistency for the species‐specific recovery of soil carbon and nutrient availabilities (Figure 4). Species with lower RN or MF tended to recover faster in ${\mathrm{PO}}_{4}^{3-}$ and ${\mathrm{NH}}_{4}^{+}$; species with lower RNSC, LN, or SLA tended to recover faster in ${\mathrm{NO}}_{3}^{-}$. None of the measured traits were significant in determining species variation in recoveries of DOC.

## 4 Discussion

Our study investigated how different subtropical woody species vary in their soil functional responses to an experimental extreme drought event. We found that fine root traits—particularly their chemical traits—played critical roles in determining plant species variation in soil functional responses to drought. Our findings have important implications for more accurately predicting the impacts of drought on species‐rich subtropical forest ecosystems.

We proposed that the responses of plant and soil components to drought are convergent across different woody species. However, our findings do not support this hypothesis. Neither habitat types nor leaf water potential at turgor loss point (TLP) can predict the resistance and recovery of soil functions of different species well. These results suggest that plant–soil drought responses may not be convergent across woody species in subtropical Danxia landform ecosystems. This non‐convergence implies that drought response patterns in plants cannot be simply extrapolated to predict rhizosphere soil functionality, thereby complicating predictions of drought impacts on species‐rich forest ecosystems. This non‐convergence may stem from the following reasons. First, the observed decoupling relationship between TLP and soil functional response to drought may stem from physiological strategies employed by drought‐tolerant plants under stress, which prioritize their survival above all else. Species with a lower TLP may follow a more conservative resource acquisition strategy (Reich 2014). Under drought stress, they may allocate limited carbon resources to critical physiological functions such as osmotic adjustment, rather than to rhizosphere carbon deposition. This may result in carbon starvation to rhizosphere microbial communities (Preece and Peñuelas 2016; Williams and de Vries 2020). In this case, the drought resistance of soil functions related to microbial communities may not be high for plants tolerant to drought. Second, beyond plant traits, other determinants may significantly influence soil functional responses to drought; for example, rhizosphere and endophytic microbial community composition and functional groups (Delgado‐Baquerizo et al. 2017; Williams and de Vries 2020; Tedersoo and Bahram 2019). While previous studies suggested mycorrhizal fungi as potential mediators of species‐specific drought responses (Mariotte et al. 2017; Jia et al. 2021), our results do not support this hypothesis, as we found no significant relationship between the proportion of mycorrhizal fungi and soil functional resistance to drought. Thus, other microbial functional groups may play more important roles in soil functional resilience in subtropical Danxia ecosystems. Third, although hilltop and valley habitats exhibit pronounced soil moisture contrasts, they are also characterized by a suite of divergent biotic and abiotic conditions. These include microclimatic factors (e.g., temperature, light intensity, wind speed), soil properties (e.g., depth, nutrient availability, texture), and distinct plant community assemblages with varying reproductive strategies and life histories (Peng et al. 2018). Consequently, plant habitat preferences likely result from species adaptation to multiple environmental factors rather than soil moisture alone. Fourth, to minimize confounding factors, we conducted the drought experiment using homogenized soil. This approach may have dampened potential microbial‐driven divergence in plant–soil responses that could emerge under natural, unhomogenized conditions. Future studies incorporating different soil types or large‐scale field experiments would help verify this possibility. Finally, the study included a limited number of species (*n* = 10), and sample sizes across habitat‐specialist groups were uneven (e.g., only 2 hilltop specialist species). This sampling structure may limit the statistical power of our tests.

Our findings demonstrate that traits with closer soil contact under ambient conditions, particularly root and chemical traits, serve as superior predictors of interspecific variation in soil functional responses to drought. Species with higher RNP and RNSC consistently recovered more slowly in all four soil enzyme activities. For species with higher RNSC, the slower soil enzyme recovery is likely related to carbon allocation dynamics induced by drought. During drought, these species with high RNSC may consume soluble sugars from RNSC reserves to maintain osmotic adjustment (O'Brien et al. 2014; Mitchell et al. 2013). This may deplete carbon substrates available for exudation during recovery and inhibit rhizosphere microbial activities and recovery of soil enzyme activities (Williams and de Vries 2020). In addition to soil functional recovery, we found that chemical traits (RP, RNP, LNP, and LNSC) and root phenotypic traits (SRA and RBI) are also important in determining interspecific variation in soil functional resistance to drought. Previous studies have identified plant traits important for predicting soil functions under ambient conditions (Wan et al. 2022, 2021; Han et al. 2023; Orwin et al. 2010; Cantarel et al. 2015), yet few studies have identified the key traits governing interspecific variation in soil functional responses to drought. It has been proposed that plant mycorrhizal association types (Phillips et al. 2013; Bardgett et al. 2014) and root phenotypic plasticity (de Vries et al. 2016) are important predictors of soil nutrient cycling under climate change. Our study introduces root chemical trait as a third, potentially key factor for predicting soil functional responses to extreme drought. However, the specific mechanisms by which these root chemical traits modulate soil functional responses to drought—through regulating root exudates and altering rhizosphere microbial community structure and function—remain unclear and warrant further investigation.

The consistent effects of RNP and RNSC on species‐specific recovery of soil enzyme activities after drought imply that root chemical traits could represent key predictors of drought resilience in forest ecosystems. However, the predictive power of plant species' root chemical traits for soil functional responses to drought may differ in field settings. For example, high soil heterogeneity in the field may weaken it by increasing within‐species trait variation (Bardgett et al. 2014), while strong drought legacy effects on microbial communities may strengthen it by tightening the root chemistry‐microbiome linkage. Therefore, future research should explore how both intra‐ and interspecific variation in plant traits predict soil functional responses to drought in more complex, heterogeneous field settings.

## 5 Conclusions

To predict how intensifying drought events will affect soil functioning in tropical and subtropical forest ecosystems, it is essential to understand how species from different moisture‐related habitats vary in their drought responses. Our study reveals that plant and soil functional responses to drought are partially decoupled, limiting the utility of plant‐level drought responses alone for predicting rhizosphere soil functionality. However, we identify fine‐root traits—particularly chemical traits—as robust predictors of interspecific variation in drought recovery of rhizosphere soil enzyme activities. Our findings suggest prioritizing plant species with lower RNP and RNSC may accelerate the post‐drought recovery of rhizosphere soil functionality in both artificial afforestation projects and the adaptive management of natural forests. These findings also significantly advance our capacity to forecast drought impacts on soil functioning in species‐rich forests.

## Author Contributions

**Lei Wang:** formal analysis (lead), investigation (lead), writing – original draft (lead). **Zewei Zhuang:** formal analysis (lead), investigation (lead), writing – original draft (lead). **Zheyang Su:** formal analysis (supporting), investigation (supporting), writing – review and editing (supporting). **Wenbin Li:** investigation (supporting), writing – review and editing (supporting). **Qizhi Wang:** investigation (supporting), writing – review and editing (supporting). **Xinfeng Chen:** investigation (supporting), writing – review and editing (supporting). **Ruiling Liu:** investigation (supporting), writing – review and editing (supporting). **Haoliang Lu:** conceptualization (supporting), writing – review and editing (supporting). **Yuxin Chen:** conceptualization (lead), formal analysis (supporting), funding acquisition (lead), supervision (lead), writing – original draft (lead).

## Funding

This work was supported by the National Natural Science Foundation of China, 32071536, 32322052. Fundamental Research Funds for the Central Universities, 20720240091.

## Conflicts of Interest

The authors declare no conflicts of interest.

## Supporting information


**Data S1:** ece373048‐sup‐0001‐Supinfo.docx.

## Data Availability

The experimental data and R code are uploaded to Figshare: https://figshare.com/s/90b66f9ca86690316aa1.
