# Supplementary material for: Root Traits Predict the Soil Functional Responses of Subtropical Plant Species to Experimental Drought
Source: Ecol Evol. 2026 Feb 3;16(2):e73048. doi: 10.1002/ece3.73048 (PMC12865509; doi:10.1002/ece3.73048)
Supplement: Supplementary file 1 — Data S1: ece373048‐sup‐0001‐Supinfo.docx. [file ECE3-16-e73048-s001.docx]

**Supporting Information**


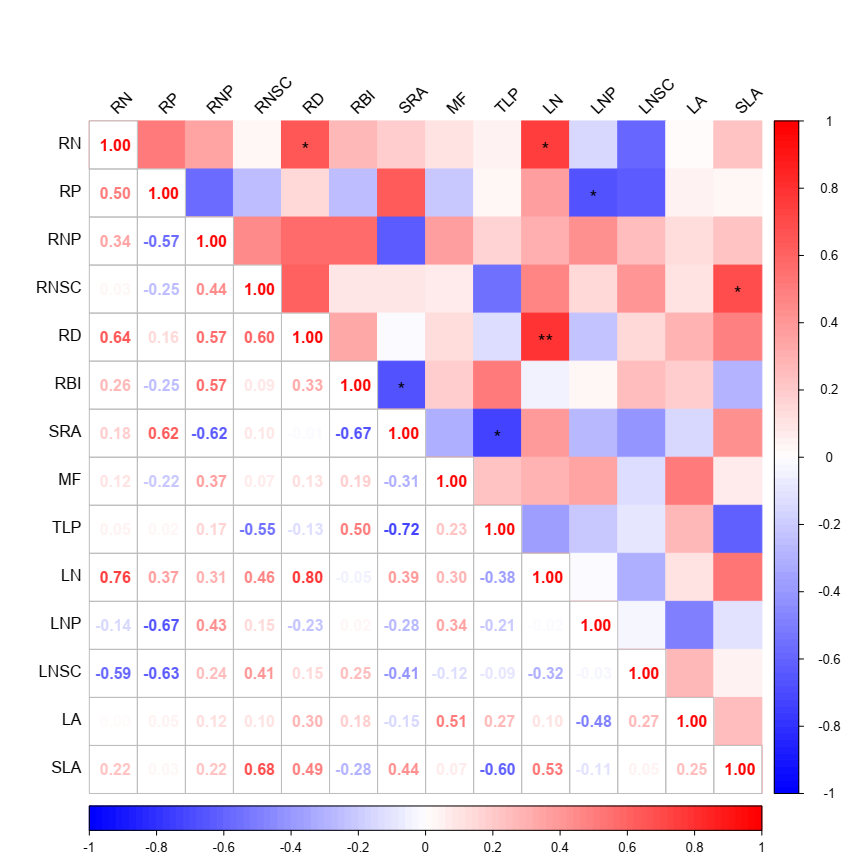


**Supplementary Figure 1 Pearson’s correlation among plant traits across species.** Significant correlation coefficients are indicated with asterisk (*, *p* < 0.05; **, *p* < 0.01). The full name of plant traits can be found in Table 2.


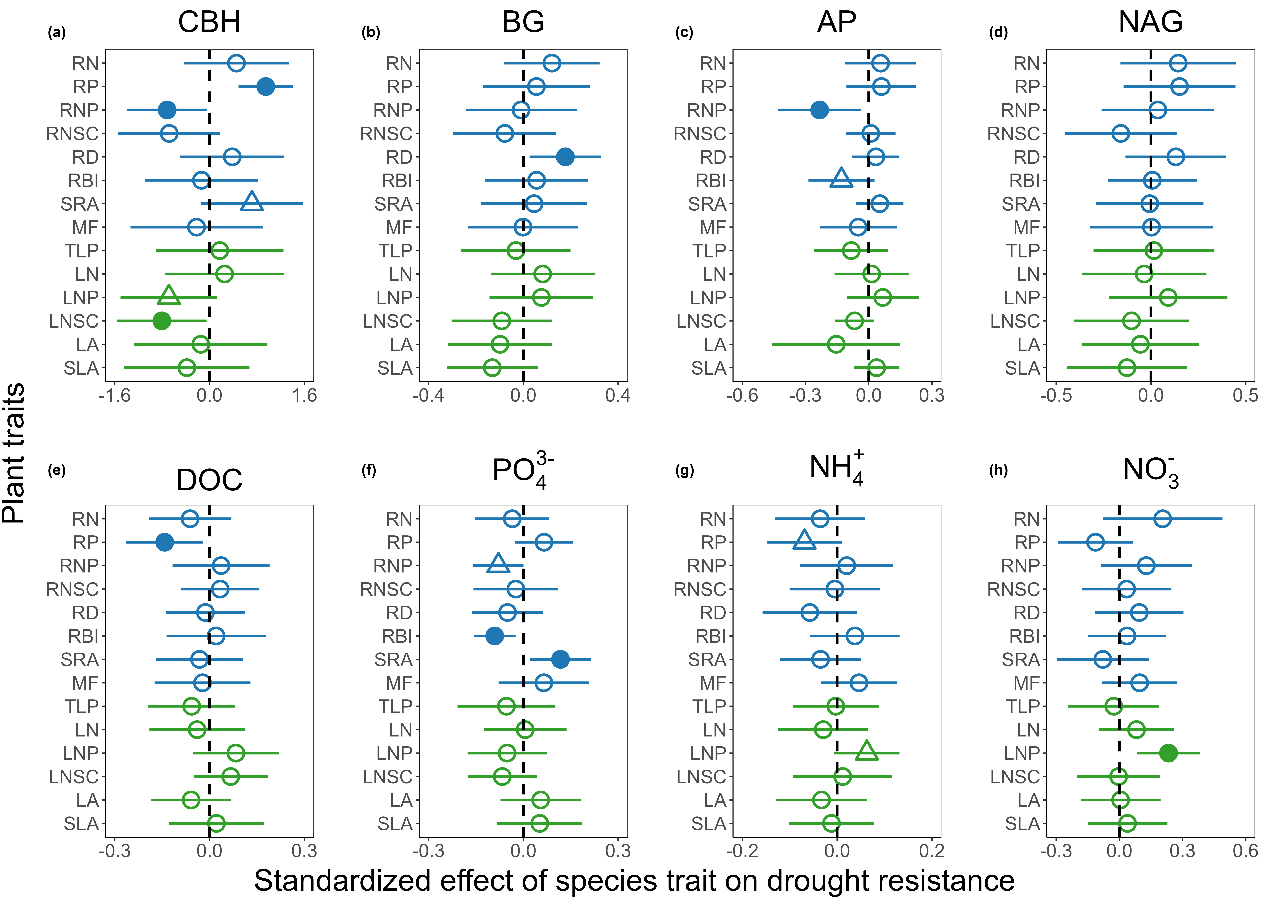


**Supplementary Figure 2 Effects of plant functional traits on species-specific resistance of rhizosphere soil enzyme activity (a–d), carbon and nutrient availability (e–h) to drought.** It is a sensitivity analysis compared to Figure 3. We repeated the analysis in Figure 3 by removing the species with the largest Cook's distance greater than 1. Soil enzyme activities include cellobiohydrolase (CBH), β-1,4-glucosidase (BG), acid phosphatase (AP), and β-1,4-*N*-acetylglucosaminidase (NAG). Soil carbon and nutrient availabilities include dissolved organic carbon (DOC), available phosphorus (PO_4_^3-^), ammonium nitrogen (NH_4_^+^), and nitrate nitrogen (NO_3_^-^). Points and lines represent mean standardized effects and their 95% confidence intervals (CIs), respectively. Blue and green symbols indicate results from fine root and leaf traits. Solid circles indicate statistically significant effects if the 95% CIs exclude zero; triangles indicate marginally significant effects if the 90% CIs exclude zero; empty circles indicate non-significant effects if the 90% CIs include zero. The full name of plant traits can be found in Table 2.


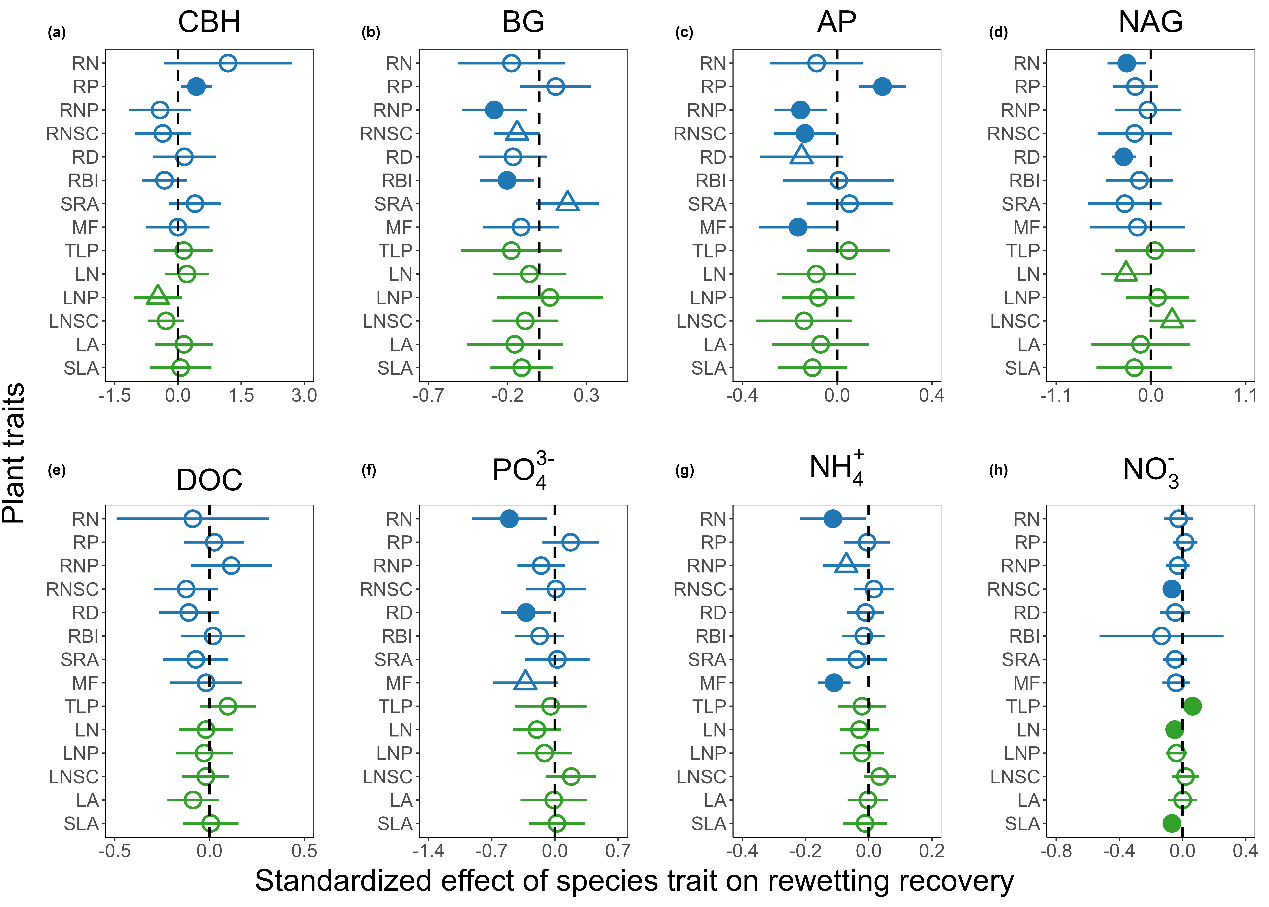


**Supplementary Figure 3 Effects of plant functional traits on species-specific recovery of rhizosphere soil enzyme activity (a–d), carbon and nutrient availability (e–h) from rewetting.** It is a sensitivity analysis compared to Figure 4. We repeated the analysis in Figure 4 by removing the species with the largest Cook's distance greater than 1. Soil enzyme activities include cellobiohydrolase (CBH), β-1,4-glucosidase (BG), acid phosphatase (AP), and β-1,4-*N*-acetylglucosaminidase (NAG). Soil carbon and nutrient availabilities include dissolved organic carbon (DOC), available phosphorus (PO_4_^3-^), ammonium nitrogen (NH_4_^+^), and nitrate nitrogen (NO_3_^-^). Points and lines represent mean standardized effects and their 95% confidence intervals (CIs), respectively. Blue and green symbols indicate results from fine root and leaf traits. Solid circles indicate statistically significant effects if the 95% CIs exclude zero; triangles indicate marginally significant effects if the 90% CIs exclude zero; empty circles indicate non-significant effects if the 90% CIs include zero. The full name of plant traits can be found in Table 2.


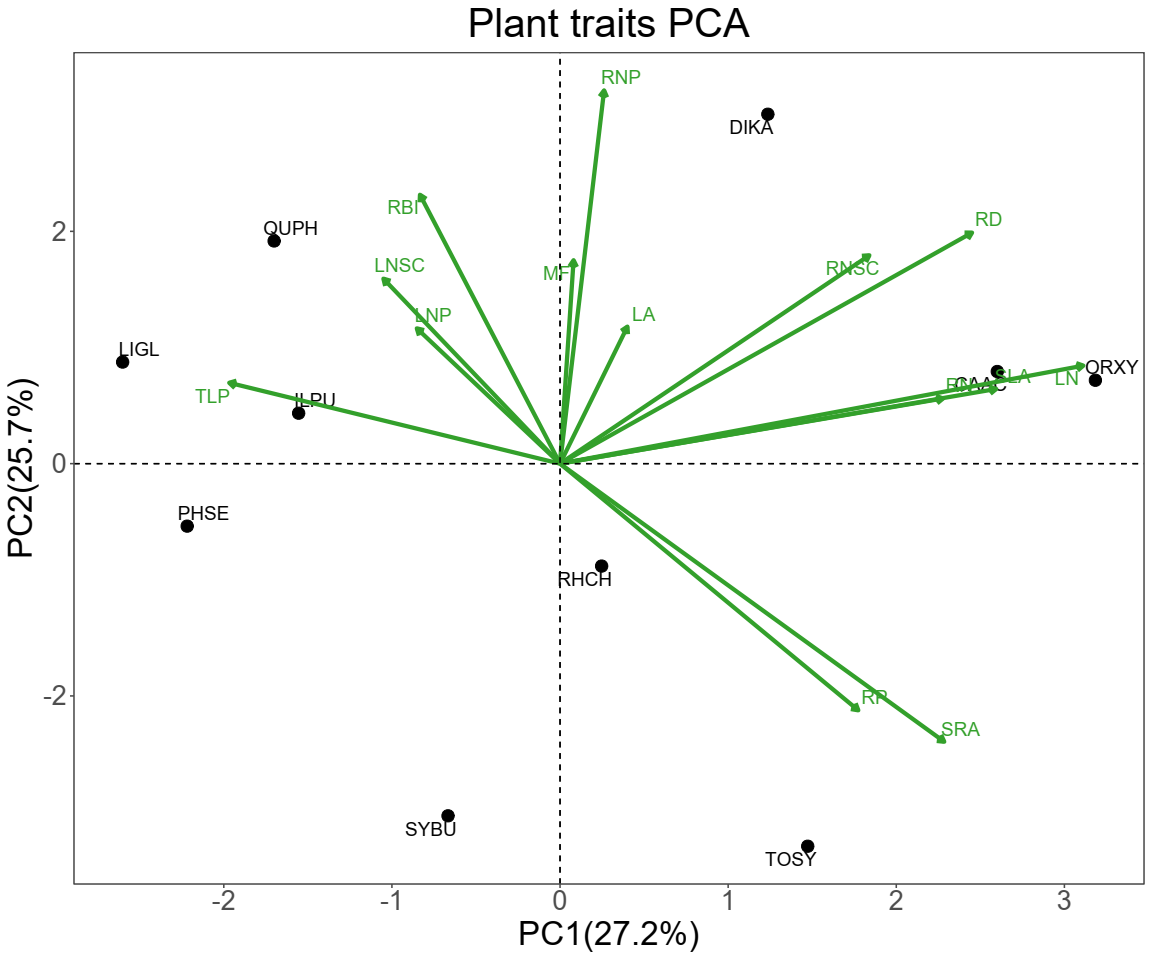


**Supplementary Figure 4** **Principal component analysis (PCA) of plant functional traits across species and their distribution in the PCA ordination.** Black circles denote species. Full species names are listed in Table 1. The full name of plant traits can be found in Table 2.


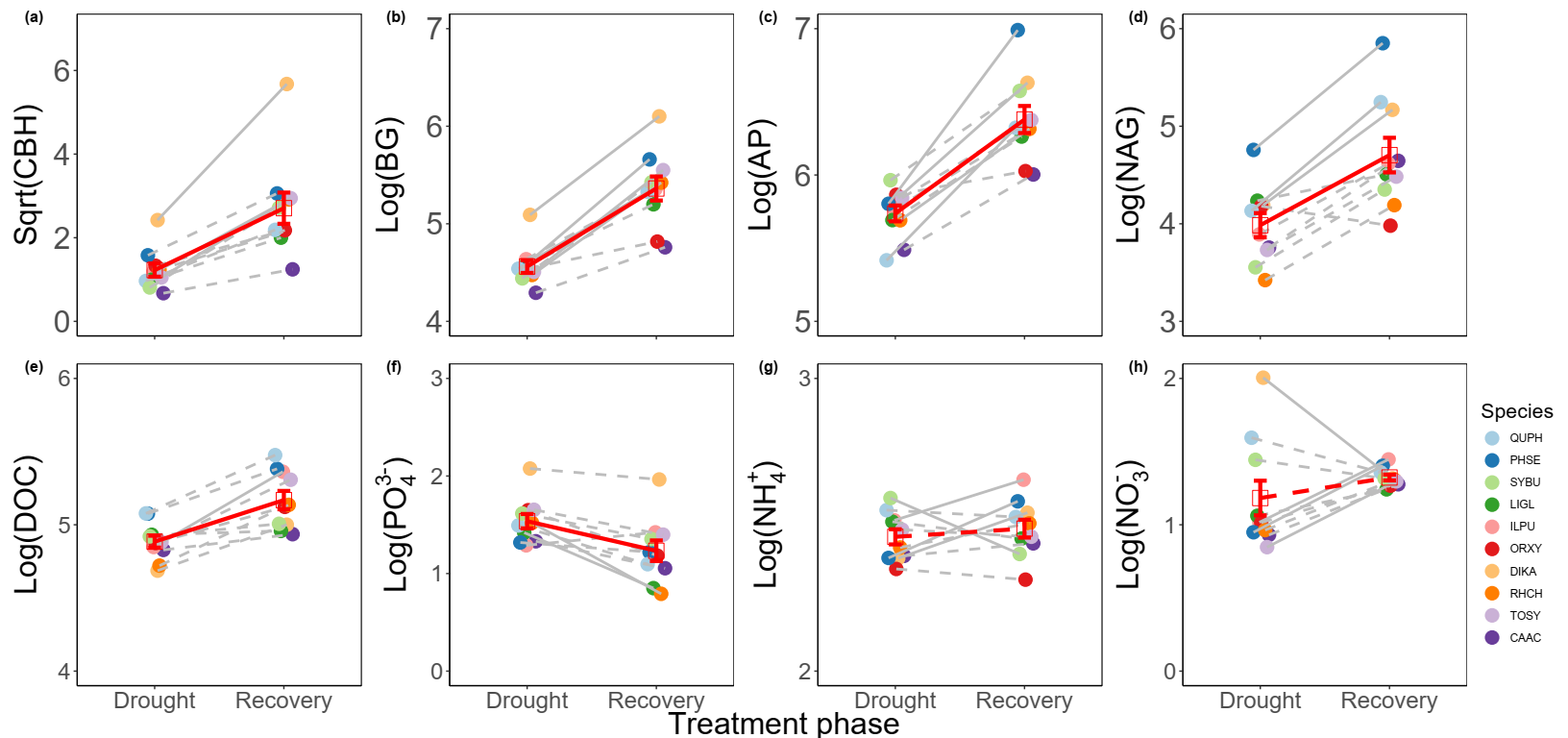


**Supplementary Figure 5 Differences in rhizosphere soil enzyme activities, and carbon and nutrient availabilities of drought-treated plants between** **drought and recovery phases**. Soil enzyme activities include cellobiohydrolase (CBH, nmol g^-1^ h^-1^, a), β-1,4-glucosidase (BG, nmol g^-1^ h^-1^, b), acid phosphatase (AP, nmol g^-1^ h^-1^, c), β-1,4-*N*-acetylglucosaminidase (NAG, nmol g^-1^ h^-1^, d). Soil carbon and nutrient availabilities include dissolved organic carbon (DOC, mg kg^-1^, e), available phosphorus (PO_4_^3-^, mg kg^-1^, f), ammonium nitrogen (NH_4_^+^, mg kg^-1^, g), and nitrate nitrogen (NO_3_^-^, mg kg^-1^, h). Red solid lines indicate significant differences in the mean values across species between drought and recovery phases, while red dashed lines indicate non-significant differences (Supplementary Table 9). Red square points with error bars represent means ± standard error of soil variables across different woody species for the corresponding phase. Solid circles indicate species-specific means. Gray solid lines indicate significant differences for individual species between drought and recovery phases, while gray dashed lines indicate non-significance. Full species names are listed in Table 1.


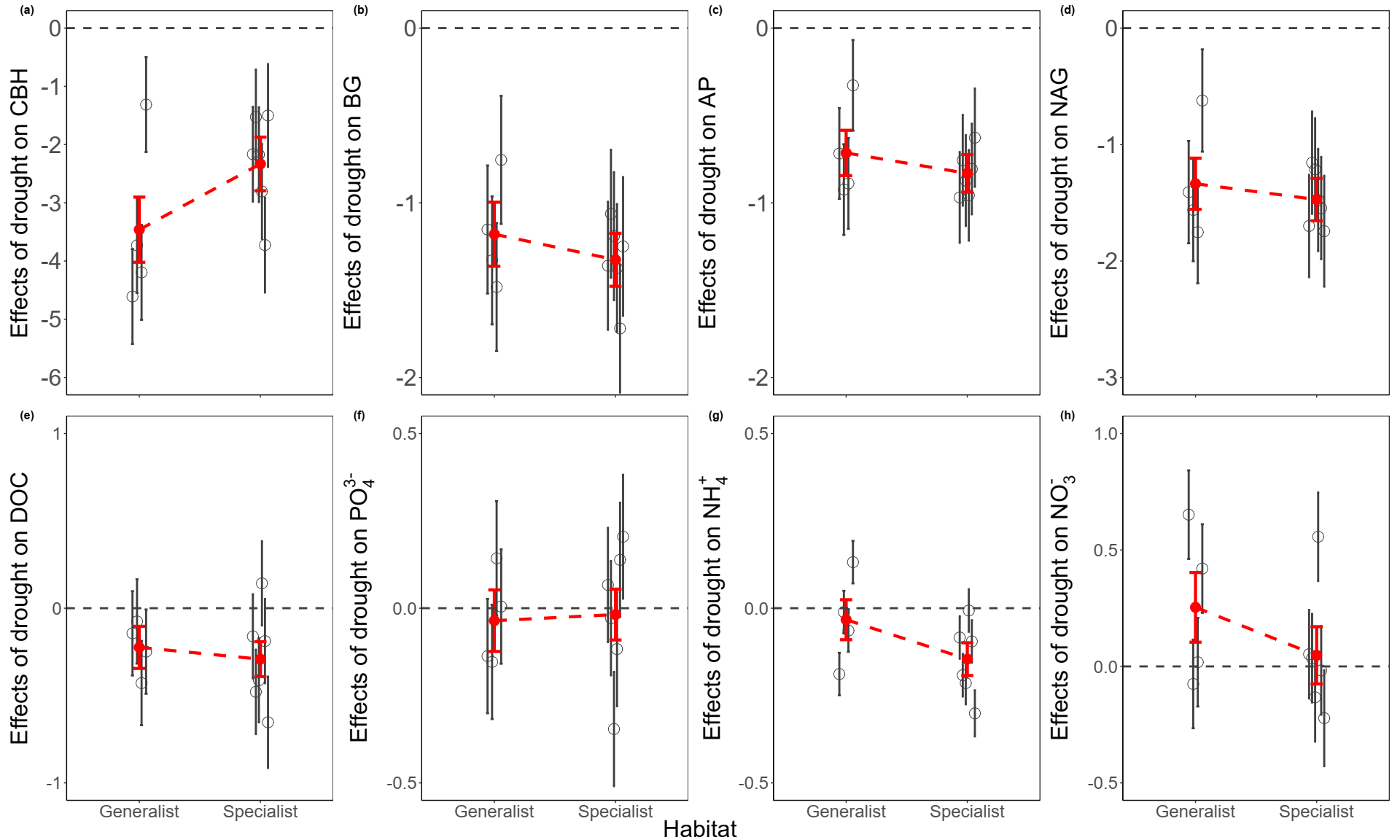


**Supplementary Figure 6 Difference in the effects of drought (end of drought) on rhizosphere soil enzyme activity, and carbon and nutrient availability between habitat generalist and specialist species.** Soil enzyme activities include cellobiohydrolase (CBH, a), β-1,4-glucosidase (BG, b), acid phosphatase (AP, c), β-1,4-*N*-acetylglucosaminidase (NAG, d). Soil carbon and nutrient availabilities include dissolved organic carbon (DOC, e), available phosphorus (PO_4_^3-^, f), ammonium nitrogen (NH_4_^+^, g), and nitrate nitrogen (NO_3_^-^, h). Red points with error bars represent means ± standard error of effects of drought on soil functions across woody species for the corresponding species group. Red solid lines indicate significant differences in the mean values between generalist and specialist species, while red dashed lines denote non-significance (Supplementary Table 9). Gray points with error bars indicate means ± standard error of drought effects per species.


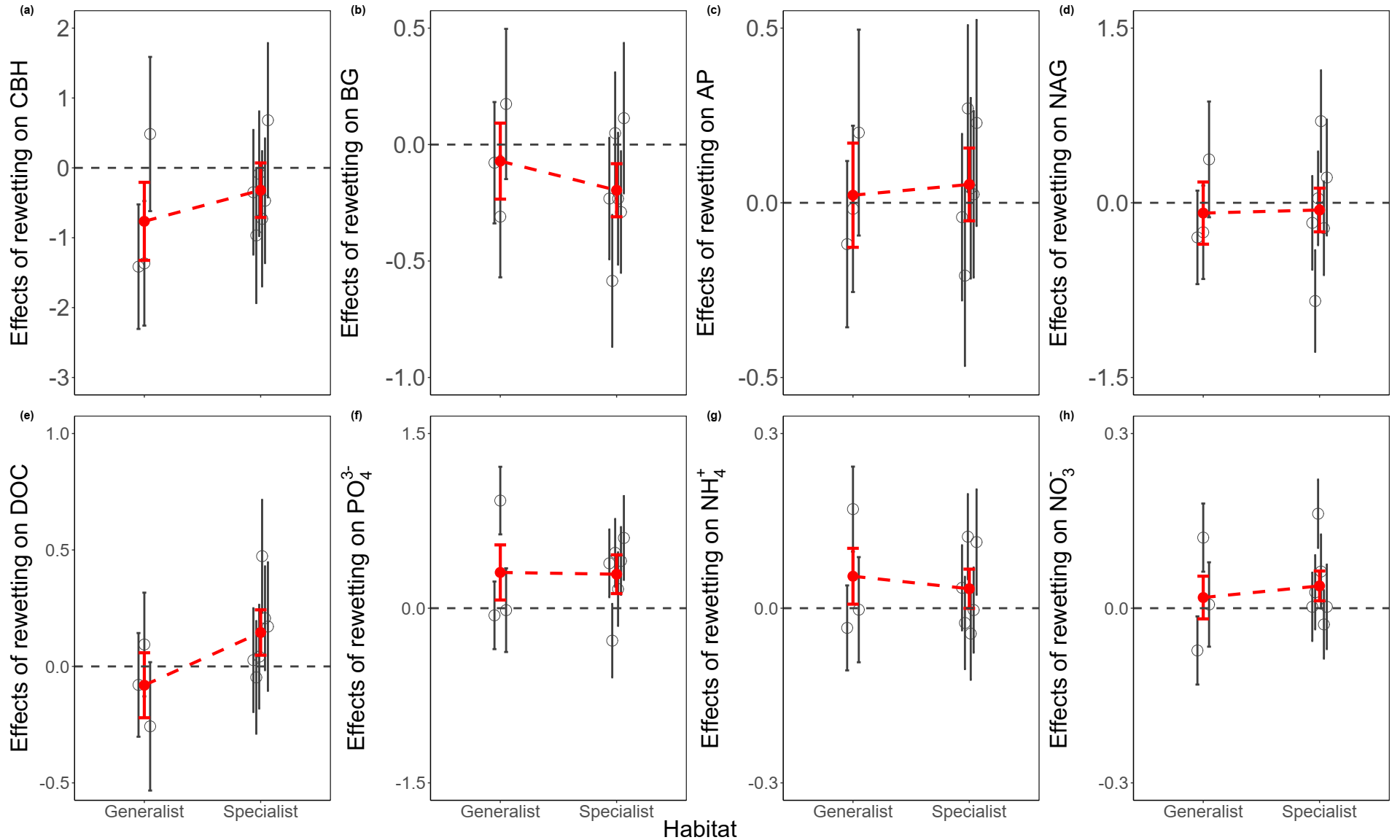


**Supplementary Figure 7** **Difference in the effects of** **rewetting after drought (end of recovery) on rhizosphere soil enzyme activity, and carbon and nutrient availability between habitat generalist and specialist species.** Soil enzyme activities include cellobiohydrolase (CBH, a), β-1,4-glucosidase (BG, b), acid phosphatase (AP, c), β-1,4-*N*-acetylglucosaminidase (NAG, d). Soil carbon and nutrient availabilities include dissolved organic carbon (DOC, e), available phosphorus (PO_4_^3-^, f), ammonium nitrogen (NH_4_^+^, g), and nitrate nitrogen (NO_3_^-^, h). Red points with error bars represent means ± standard error of effects of rewetting on soil functions across woody species for the corresponding species group. Red solid lines indicate significant differences in the mean values between generalist and specialist species, while red dashed lines denote non-significance (Supplementary Table 10). Gray points with error bars indicate means ± standard error of rewetting effects per species. Data for ambient treatment of species *Lithocarpus glaber* (LIGL) during recovery phase are missing due to insufficient plant samples.


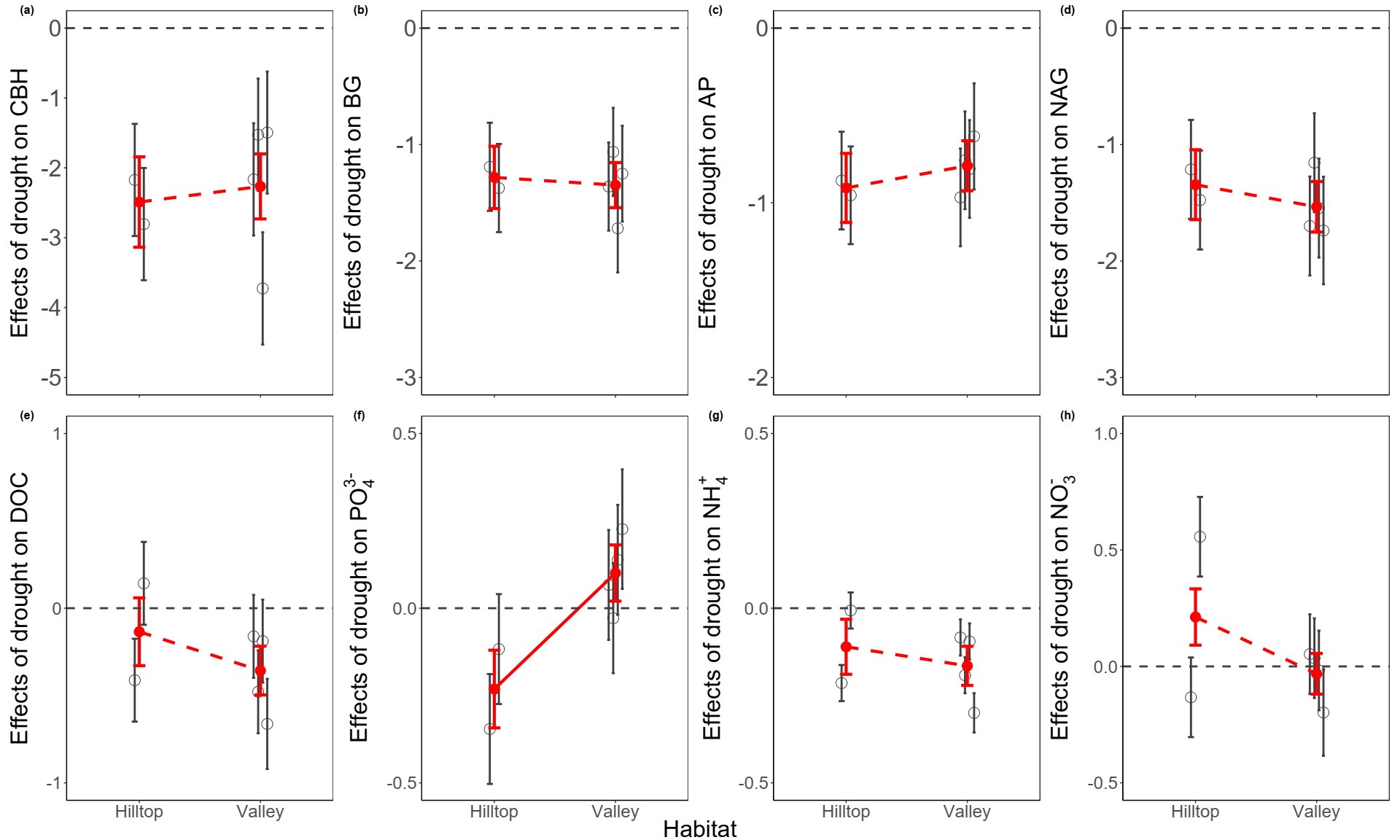


**Supplementary Figure 8** **Difference in the effects of drought (end of drought) on rhizosphere soil enzyme activity, and carbon and nutrient availability between species specialized in hilltop vs. valley.** Soil enzyme activities include cellobiohydrolase (CBH, a), β-1,4-glucosidase (BG, b), acid phosphatase (AP, c), β-1,4-*N*-acetylglucosaminidase (NAG, d). Soil carbon and nutrient availabilities include dissolved organic carbon (DOC, e), available phosphorus (PO_4_^3-^, f), ammonium nitrogen (NH_4_^+^, g), and nitrate nitrogen (NO_3_^-^, h). Red points with error bars represent means ± standard error of effects of drought on soil functions across woody species for the corresponding species group. Red solid lines indicate significant differences in the mean values between hilltop and valley specialists, while red dashed lines denote non-significance (Supplementary Table 11). Gray points with error bars indicate means ± standard error of drought effects per species.


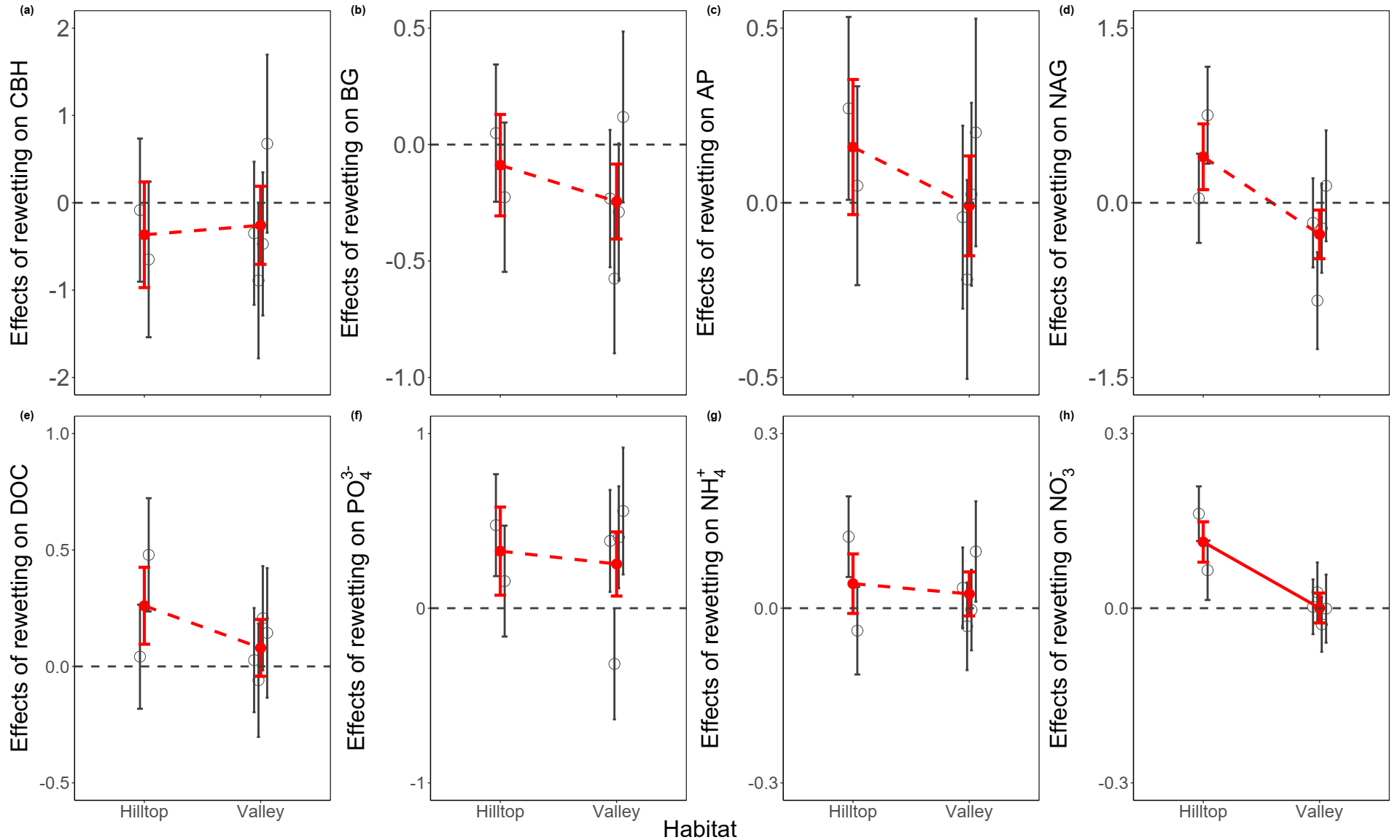


**Supplementary Figure 9** **Difference in the effects of rewetting after drought (end of recovery) on rhizosphere soil enzyme activity, and carbon and nutrient availability between species specialized in hilltop vs. valley.** Soil enzyme activities include cellobiohydrolase (CBH, a), β-1,4-glucosidase (BG, b), acid phosphatase (AP, c), β-1,4-*N*-acetylglucosaminidase (NAG, d). Soil carbon and nutrient availabilities include dissolved organic carbon (DOC, e), available phosphorus (PO_4_^3-^, f), ammonium nitrogen (NH_4_^+^, g), and nitrate nitrogen (NO_3_^-^, h). Red points with error bars represent means ± standard error of effects of rewetting on soil functions across woody species for the corresponding species group. Red solid lines indicate significant differences in the mean values across species between hilltop and valley specialists, while red dashed lines denote non-significance (Supplementary Table 11). Gray points with error bars indicate means ± standard error of rewetting effects per species. Data for ambient treatment of tree species *Lithocarpus glaber* (LIGL) during recovery phase are missing due to insufficient plant samples.


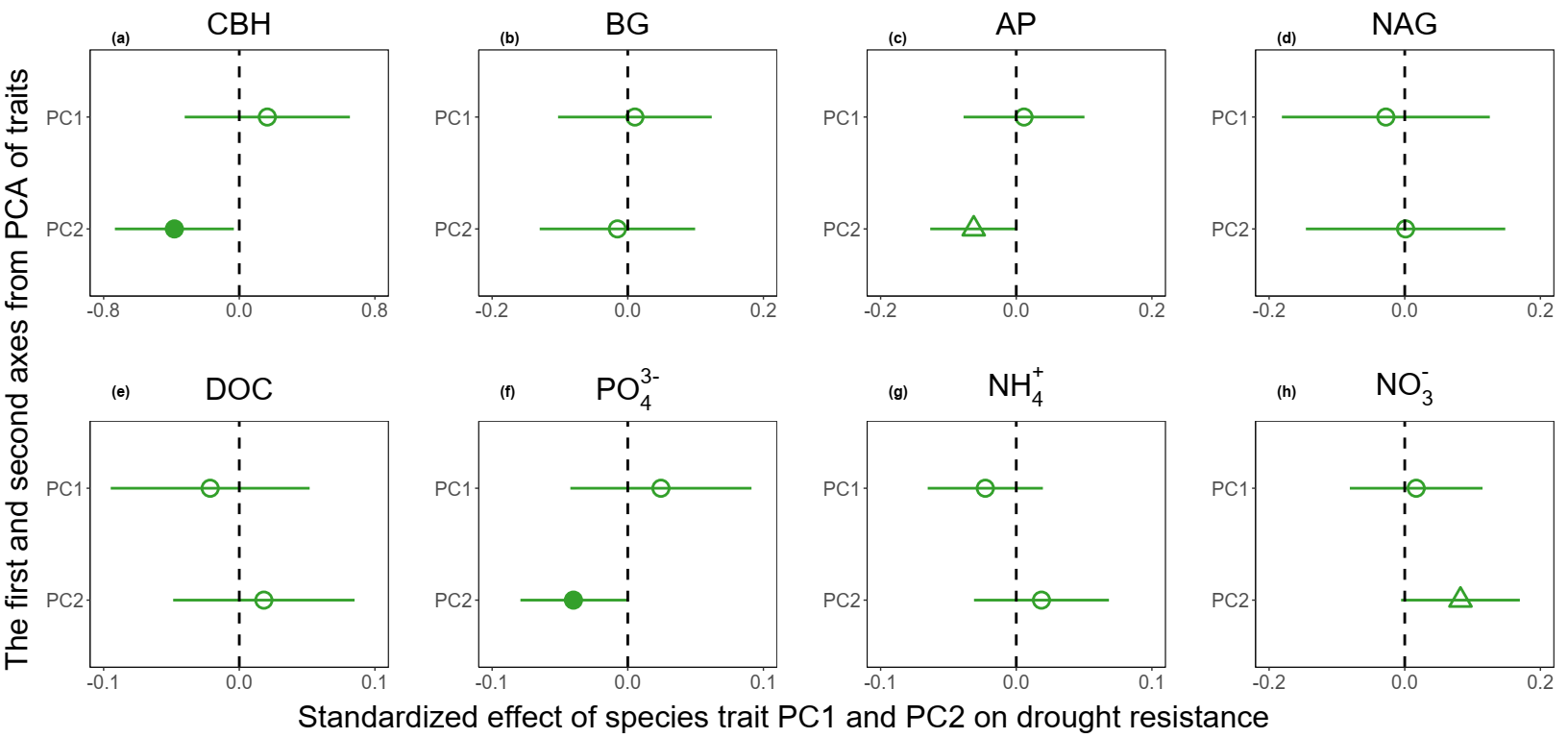


**Supplementary Figure 10 Effects of multiple species trait (PC1 and PC2) on species-specific resistance of rhizosphere soil enzyme activity (a–d), carbon and nutrient availability (e–h) to drought.** Soil enzyme activities include cellobiohydrolase (CBH), β-1,4-glucosidase (BG), acid phosphatase (AP), and β-1,4-*N*-acetylglucosaminidase (NAG). Soil carbon and nutrient availabilities include dissolved organic carbon (DOC), available phosphorus (PO_4_^3-^), ammonium nitrogen (NH_4_^+^), and nitrate nitrogen (NO_3_^-^). Points and lines represent mean standardized effects and their 95% confidence intervals (CIs), respectively. Solid circles indicate statistically significant effects if the 95% CIs exclude zero; triangles indicate marginally significant effects if the 90% CIs exclude zero; empty circles indicate non-significant effects.


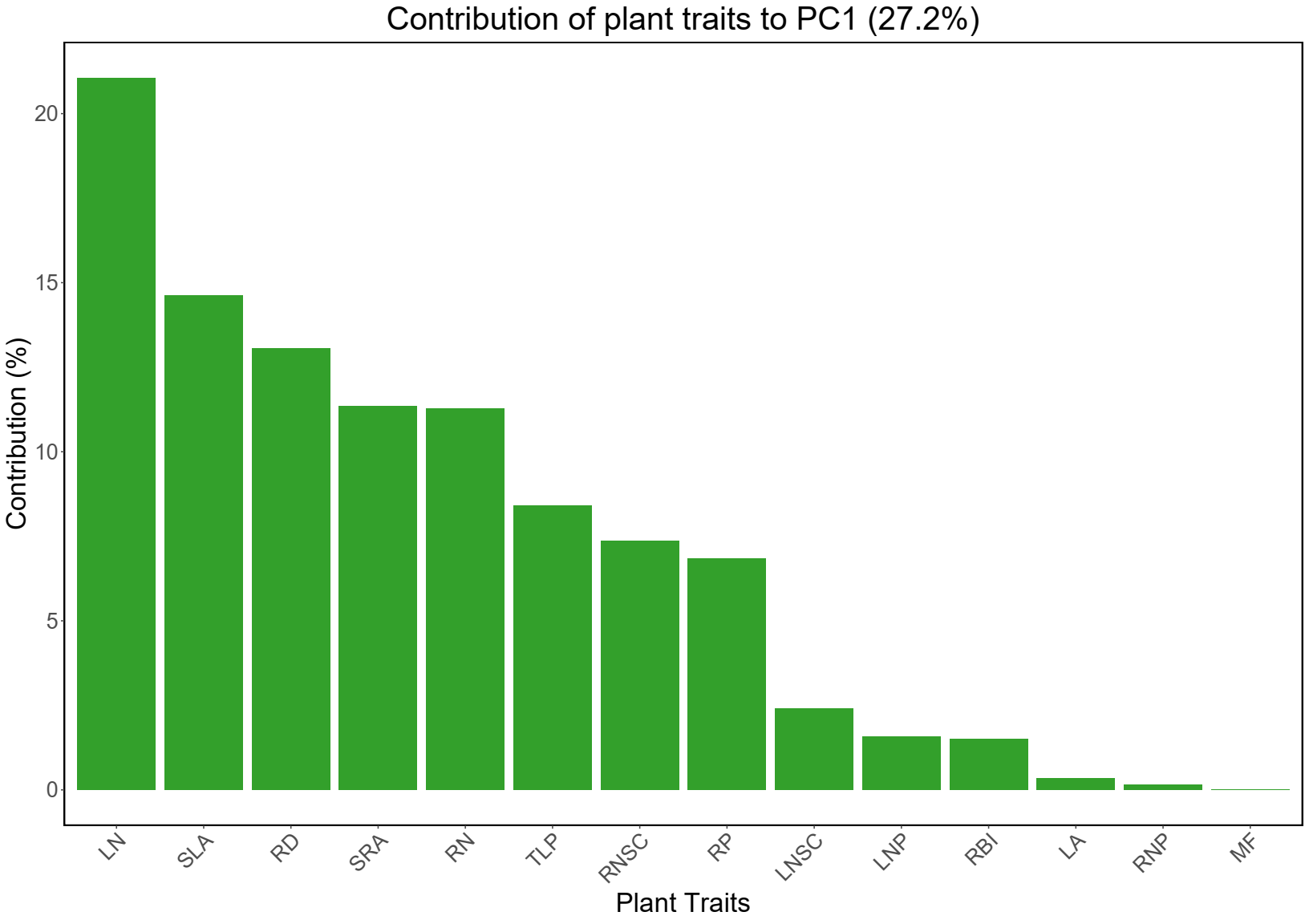


**Supplementary Figure 11 Contribution of plant traits to the first axis from PCA of plant traits.** The full name of plant traits can be found in Table 2.


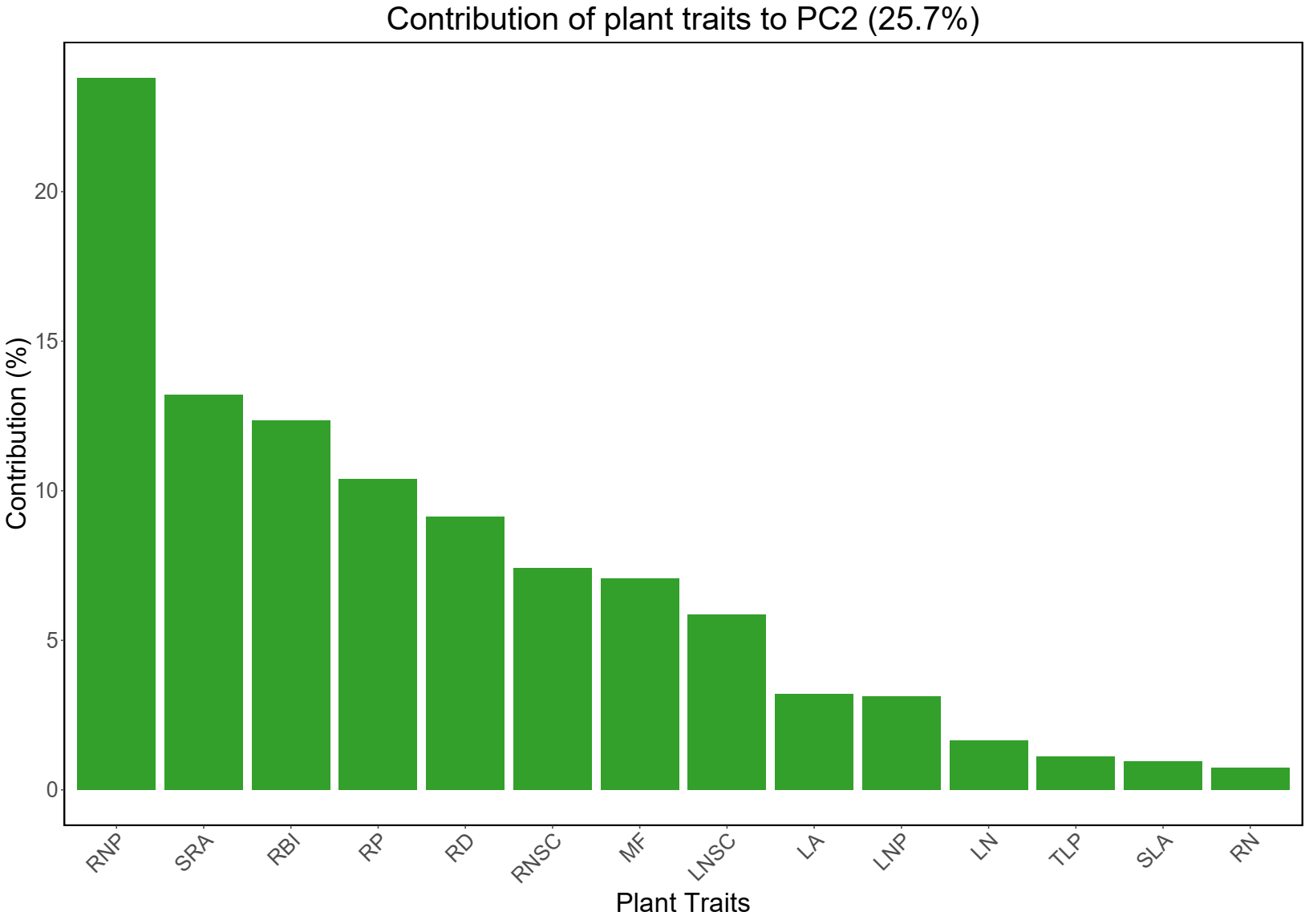


**Supplementary Figure 12 Contribution of plant traits to the second axis from PCA of plant traits.** The full name of plant traits can be found in Table 2.


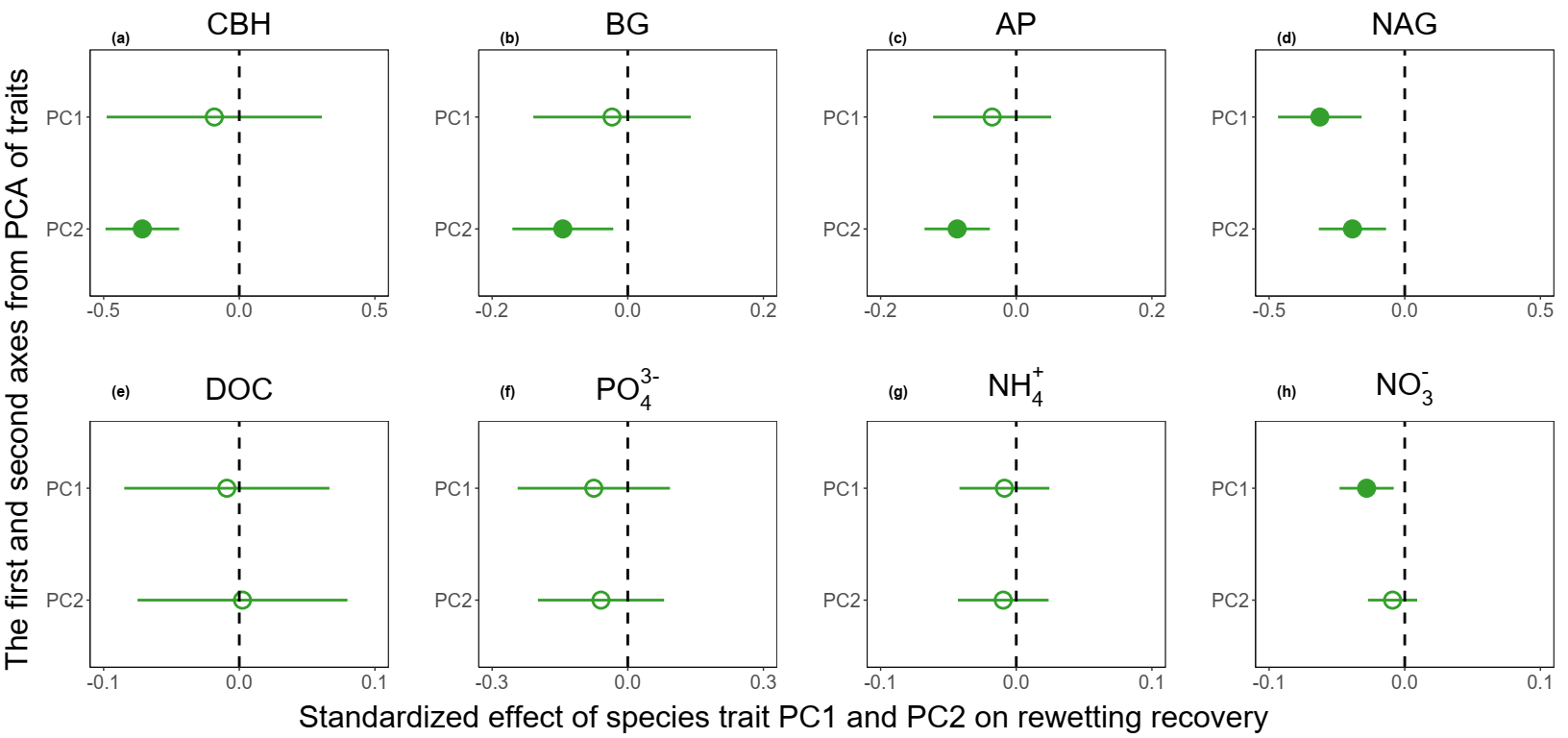


**Supplementary Figure 13 Effects of multiple species trait** **(PC1 and PC2) on species-specific recovery of rhizosphere soil enzyme activity (a–d), carbon and nutrient availability (e–h) to** **rewetting.** Soil enzyme activities include cellobiohydrolase (CBH), β-1,4-glucosidase (BG), acid phosphatase (AP), and β-1,4-*N*-acetylglucosaminidase (NAG). Soil carbon and nutrient availabilities include dissolved organic carbon (DOC), available phosphorus (PO_4_^3-^), ammonium nitrogen (NH_4_^+^), and nitrate nitrogen (NO_3_^-^). Points and lines represent mean standardized effects and their 95% confidence intervals (CIs), respectively. Solid circles indicate statistically significant effects if the 95% CIs exclude zero; empty circles indicate non-significant effects.

**Supplementary Table 1** Sample sizes of soil functions per species in different experimental phases and treatments. The scales at which we seek to make inferences are at the unit of treatment (ambient vs. drought) and species. We applied the treatment at the level of plant individual.

|  |  | **End of drought** | | **End of recovery** | |
| --- | --- | --- | --- | --- | --- |
| **Species** | **Abbreviation** | **Ambient** | **Drought** | **Ambient** | **Drought** |
| *Quercus phillyraeoides* | QUPH | 4 | 4 | 3 | 4 |
| *Photinia serratifolia* | PHSE | 4 | 4 | 4 | 4 |
| *Syzygium buxifolium* | SYBU | 4 | 4 | 4 | 2 |
| *Lithocarpus glaber* | LIGL | 4 | 4 | 0 | 3 |
| *Ilex pubescens* | ILPU | 4 | 4 | 4 | 4 |
| *Ormosia xylocarpa* | ORXY | 4 | 4 | 4 | 3 |
| *Diospyros kaki* var*.* | DIKA | 4 | 4 | 4 | 4 |
| *Rhus chinensis* | RHCH | 4 | 4 | 4 | 4 |
| *Toxicodendron sylvestre* | TOSY | 3 | 4 | 2 | 4 |
| *Camptotheca acuminata* | CAAC | 4 | 4 | 4 | 4 |
| Total |  | 39 | 40 | 33 | 36 |

**Supplementary Table 2** Sample sizes of plant functional traits per species under ambient conditions. The scales at which we seek to make inferences are at the unit of species.

| **Species** | **Abbreviation** | **RN** | **RP** | **RNP** | **RNSC** | **RD** | **RBI** | **SRA** | **MF** | **TLP** | **LN** | **LNP** | **LNSC** | **LA** | **SLA** |
| --- | --- | --- | --- | --- | --- | --- | --- | --- | --- | --- | --- | --- | --- | --- | --- |
| *Quercus phillyraeoides* | QUPH | 3 | 3 | 3 | 3 | 3 | 3 | 3 | 3 | 4 | 3 | 3 | 3 | 4 | 5 |
| *Photinia serratifolia* | PHSE | 4 | 4 | 4 | 4 | 4 | 4 | 4 | 4 | 4 | 4 | 4 | 4 | 4 | 5 |
| *Syzygium buxifolium* | SYBU | 3 | 3 | 2 | 3 | 4 | 4 | 4 | 4 | 4 | 3 | 3 | 4 | 4 | 8 |
| *Lithocarpus glaber* | LIGL | 4 | 4 | 4 | 4 | 4 | 4 | 4 | 4 | 4 | 4 | 4 | 4 | 4 | 4 |
| *Ilex pubescens* | ILPU | 4 | 4 | 4 | 4 | 4 | 4 | 4 | 4 | 4 | 4 | 4 | 4 | 4 | 7 |
| *Ormosia xylocarpa* | ORXY | 4 | 4 | 4 | 4 | 4 | 4 | 4 | 4 | 4 | 4 | 4 | 4 | 4 | 5 |
| *Diospyros kaki* var*.* | DIKA | 4 | 4 | 4 | 4 | 4 | 4 | 4 | 4 | 2 | 4 | 4 | 4 | 3 | 6 |
| *Rhus chinensis* | RHCH | 4 | 4 | 4 | 4 | 4 | 4 | 4 | 4 | 4 | 4 | 4 | 4 | 4 | 7 |
| *Toxicodendron sylvestre* | TOSY | 3 | 3 | 3 | 3 | 4 | 3 | 3 | 4 | 3 | 3 | 3 | 3 | 3 | 5 |
| *Camptotheca acuminata* | CAAC | 4 | 4 | 4 | 4 | 4 | 4 | 4 | 4 | 4 | 4 | 4 | 4 | 4 | 7 |

**Supplementary Table 3** Significance tests of drought effects on rhizosphere soil functions.

| **Soil function** | ***df*** | ***ddf*** | ***F*** | **Ambient** | **Drought** | **Treatment effect** | ***P*** |
| --- | --- | --- | --- | --- | --- | --- | --- |
| CBH(n=79) | 1 | 9 | 53.20 | 3.99(0.48) | 1.22(0.16) | $-$2.77(0.88) | **0.001** |
| BG(n=79) | 1 | 9 | 238.09 | 5.82(0.10) | 4.56(0.07) | $-$1.26(0.40) | **0.001** |
| AP(n=79) | 1 | 9 | 162.66 | 6.52(0.04) | 5.74(0.05) | $-$0.78(0.25) | **0.001** |
| NAG(n=79) | 1 | 9 | 165.94 | 5.39(0.17) | 3.99(0.12) | $-$1.41(0.44) | **0.001** |
| DOC(n=79) | 1 | 9 | 13.06 | 5.15(0.08) | 4.88(0.04) | $-$0.27(0.08) | **0.006** |
| PO_4_^3-^(n=79) | 1 | 9 | 0.24 | 1.56(0.09) | 1.54(0.07) | $-$0.02(0.01) | 0.637 |
| NH_4_^+^(n=79) | 1 | 9 | 6.46 | 2.56(0.03) | 2.46(0.03) | $-$0.10(0.03) | **0.032** |
| NO_3_^-^(n=79) | 1 | 9 | 1.98 | 1.05(0.04) | 1.18(0.12) | 0.13(0.04) | 0.193 |

Note: CBH: cellobiohydrolase; BG: β-1,4-glucosidase; AP: acid phosphatase; NAG: β-1,4-*N*- acetylglucosaminidase; DOC: dissolved organic carbon; PO_4_^3-^: available phosphorus; NH_4_^+^: ammonium nitrogen; NO_3_^-^: nitrate nitrogen. *df*, numerator degrees of freedom; *ddf*, denominator degrees of freedom (these reflect residual degrees of freedom for drought effects calculations across 10 species). *F* and *P* indicate *F* ratios and the *P* values of the significance tests, respectively. Numbers in parentheses after soil functions indicate sample sizes. Ambient and Drought show mean values (± standard error) for ambient and drought-treated groups, respectively. Treatment effect show mean values (± standard error) for the difference in soil functions between drought and ambient treatments. Significant *P* values (*P* < 0.05) are highlighted in boldface.

**Supplementary Table 4** Significance tests of rewetting effects on rhizosphere soil functions.

| **Soil function** | ***df*** | ***ddf*** | ***F*** | **Ambient** | **Drought** | **Treatment effect** | ***P*** |
| --- | --- | --- | --- | --- | --- | --- | --- |
| CBH(n=71) | 1 | 8 | 5.17 | 3.28(0.52) | 2.71(0.37) | $-$0.50(0.16) | 0.053 |
| BG(n=71) | 1 | 8 | 4.72 | 5.54(0.11) | 5.36(0.12) | $-$0.16(0.05) | 0.062 |
| AP(n=71) | 1 | 8 | 0.34 | 6.36(0.08) | 6.38(0.10) | 0.04(0.01) | 0.574 |
| NAG(n=71) | 1 | 8 | 0.30 | 4.78(0.19) | 4.70(0.18) | $-$0.06(0.02) | 0.600 |
| DOC(n=71) | 1 | 8 | 1.40 | 5.11(0.05) | 5.17(0.06) | 0.09(0.03) | 0.271 |
| PO_4_^3-^(n=71) | 1 | 8 | 5.88 | 1.00(0.18) | 1.23(0.11) | 0.28(0.09) | **0.042** |
| NH_4_^+^(n=71) | 1 | 8 | 1.96 | 2.47(0.02) | 2.49(0.03) | 0.02(0.01) | 0.199 |
| NO_3_^-^(n=71) | 1 | 8 | 1.65 | 1.31(0.01) | 1.32(0.02) | 0.03(0.01) | 0.235 |

Note: Full names of the soil function can be found in supplementary table 1. Data for ambient treatment of tree species *Lithocarpus glaber* (LIGL) during recovery phase are missing due to insufficient plant samples. *df*, numerator degrees of freedom; *ddf*, denominator degrees of freedom (these reflect residual degrees of freedom for recovery treatment effects calculations across 10 species). *F* and *P* indicate *F* ratios and the *P* values of the significance tests, respectively. Numbers in parentheses after soil functions indicate sample sizes. Ambient and Drought show mean values (± standard error) for ambient and drought-treated groups, respectively. Treatment effects show mean values (± standard error) for the differences in soil functions between drought and ambient treatments. Significant *P* values (*P* < 0.05) are highlighted in boldface.

**Supplementary Table 5** Significance tests of drought effects on rhizosphere soil enzyme activity per species.

| **Soil enzyme activity** | **Species** | **Ambient** | **Drought** | ***P*** |
| --- | --- | --- | --- | --- |
| CBH | QUPH | 3.77(0.68) | 0.97(0.34) | **0.001** |
|  | PHSE | 3.75(0.72) | 1.58(0.51) | **0.010** |
|  | SYBU | 2.12(0.79) | 0.81(0.19) | 0.112 |
|  | LIGL | 5.19(0.84) | 1.00(0.36) | **<0.0001** |
|  | ILPU | 4.93(0.40) | 1.20(0.34) | **<0.0001** |
|  | ORXY | 2.85(0.52) | 1.33(0.29) | 0.066 |
|  | DIKA | 7.03(0.49) | 2.42(0.79) | **<0.0001** |
|  | RHCH | 4.95(0.79) | 1.23(0.45) | **<0.0001** |
|  | TOSY | 2.49(0.70) | 1.05(0.36) | 0.095 |
|  | CAAC | 2.84(0.80) | 0.67(0.27) | **0.010** |
| BG | QUPH | 5.91(0.02) | 4.54(0.28) | **0.000** |
|  | PHSE | 5.72(0.10) | 4.53(0.37) | **0.002** |
|  | SYBU | 5.19(0.44) | 4.44(0.22) | **0.044** |
|  | LIGL | 6.04(0.15) | 4.56(0.15) | **0.001** |
|  | ILPU | 5.97(0.06) | 4.64(0.25) | **0.001** |
|  | ORXY | 5.61(0.18) | 4.54(0.52) | **0.005** |
|  | DIKA | 6.24(0.10) | 5.09(0.35) | **0.003** |
|  | RHCH | 6.19(0.13) | 4.48(0.20) | **<0.0001** |
|  | TOSY | 5.67(0.25) | 4.51(0.34) | **0.003** |
|  | CAAC | 5.65(0.30) | 4.29(0.25) | **0.001** |
| AP | QUPH | 6.37(0.23) | 5.42(0.28) | **0.001** |
|  | PHSE | 6.68(0.16) | 5.80(0.31) | **0.001** |
|  | SYBU | 6.29(0.34) | 5.96(0.19) | 0.212 |
|  | LIGL | 6.58(0.09) | 5.69(0.18) | **0.001** |
|  | ILPU | 6.69(0.10) | 5.77(0.10) | **0.001** |
|  | ORXY | 6.62(0.06) | 5.86(0.24) | **0.005** |
|  | DIKA | 6.56(0.05) | 5.84(0.18) | **0.008** |
|  | RHCH | 6.50(0.09) | 5.69(0.23) | **0.003** |
|  | TOSY | 6.43(0.21) | 5.84(0.21) | **0.030** |
|  | CAAC | 6.46(0.22) | 5.49(0.13) | **0.000** |
| NAG | QUPH | 5.61(0.08) | 4.13(0.18) | **0.001** |
|  | PHSE | 5.97(0.17) | 4.76(0.42) | **0.008** |
|  | SYBU | 4.18(0.58) | 3.55(0.33) | 0.162 |
|  | LIGL | 5.99(0.32) | 4.24(0.26) | **0.000** |
|  | ILPU | 5.45(0.27) | 3.89(0.19) | **0.001** |
|  | ORXY | 5.34(0.37) | 4.18(0.60) | **0.011** |
|  | DIKA | 5.60(0.28) | 4.19(0.45) | **0.002** |
|  | RHCH | 4.97(0.29) | 3.42(0.31) | **0.001** |
|  | TOSY | 5.37(0.41) | 3.73(0.43) | **0.001** |
|  | CAAC | 5.46(0.32) | 3.76(0.23) | **0.000** |

Note: Full names of the soil enzyme activities can be found in supplementary table 1. Ambient and Drought indicate mean enzyme activities (± standard error) for ambient and drought-treated groups, respectively. Significant *P* values (*P* < 0.05) are boldfaced. Full species names can be found in Table 1.

**Supplementary Table 6** Significance tests of drought effects on rhizosphere soil carbon and nutrient availability per species.

| **Soil carbon and nutrient availability** | **Species** | **Ambient** | **Drought** | ***P*** |
| --- | --- | --- | --- | --- |
| DOC | QUPH | 4.94(0.16) | 5.08 (0.13) | 0.557 |
|  | PHSE | 5.49(0.24) | 5.08 (0.09) | 0.093 |
|  | SYBU | 5.17(0.05) | 4.92 (0.02) | 0.307 |
|  | LIGL | 5.36(0.15) | 4.93 (0.12) | 0.081 |
|  | ILPU | 4.92(0.26) | 4.85 (0.15) | 0.754 |
|  | ORXY | 5.35(0.12) | 4.88 (0.05) | 0.052 |
|  | DIKA | 4.83(0.39) | 4.69 (0.04) | 0.553 |
|  | RHCH | 4.91(0.21) | 4.72 (0.11) | 0.438 |
|  | TOSY | 5.55(0.30) | 4.88 (0.19) | **0.015** |
|  | CAAC | 4.99(0.22) | 4.83 (0.16) | 0.506 |
| PO_4_^3-^ | QUPH | 1.61(0.17) | 1.49(0.18) | 0.477 |
|  | PHSE | 1.66(0.15) | 1.32(0.06) | **0.039** |
|  | SYBU | 1.61(0.18) | 1.62(0.11) | 0.977 |
|  | LIGL | 1.28(0.08) | 1.42(0.18) | 0.386 |
|  | ILPU | 1.44(0.02) | 1.29(0.17) | 0.351 |
|  | ORXY | 1.68(0.07) | 1.65(0.01) | 0.862 |
|  | DIKA | 2.21(0.04) | 2.08(0.09) | 0.406 |
|  | RHCH | 1.38(0.08) | 1.52(0.09) | 0.401 |
|  | TOSY | 1.44(0.14) | 1.65(0.12) | 0.253 |
|  | CAAC | 1.26(0.11) | 1.33(0.02) | 0.686 |
| NH_4_^+^ | QUPH | 2.56(0.03) | 2.55(0.03) | 0.913 |
|  | PHSE | 2.60(0.03) | 2.39(0.04) | **0.001** |
|  | SYBU | 2.46(0.07) | 2.59(0.10) | **0.034** |
|  | LIGL | 2.57(0.03) | 2.51(0.06) | 0.297 |
|  | ILPU | 2.53(0.05) | 2.52(0.01) | 0.856 |
|  | ORXY | 2.54(0.05) | 2.35(0.03) | **0.003** |
|  | DIKA | 2.57(0.04) | 2.39(0.04) | **0.003** |
|  | RHCH | 2.60(0.03) | 2.42(0.01) | 0.123 |
|  | TOSY | 2.78(0.09) | 2.48(0.03) | **<0.000** |
|  | CAAC | 2.48(0.08) | 2.39(0.02) | 0.173 |
| NO_3_^-^ | QUPH | 1.04(0.06) | 1.59(0.35) | **0.005** |
|  | PHSE | 1.08(0.09) | 0.95(0.06) | 0.488 |
|  | SYBU | 1.02(0.07) | 1.44(0.33) | **0.031** |
|  | LIGL | 1.04(0.08) | 1.06(0.08) | 0.925 |
|  | ILPU | 1.09(0.04) | 1.02(0.05) | 0.694 |
|  | ORXY | 0.98(0.07) | 1.02(0.15) | 0.853 |
|  | DIKA | 1.35(0.11) | 2.01(0.17) | **0.001** |
|  | RHCH | 0.98(0.11) | (0.96)0.06 | 0.928 |
|  | TOSY | 1.05(0.06) | (0.85)0.06 | 0.286 |
|  | CAAC | 0.88(0.05) | 0.93(0.07) | 0.783 |

Note: Full names of the soil carbon and nutrient availabilities can be found in supplementary table 1. Ambient and Drought indicate mean carbon and nutrient availabilities (± standard error) for ambient and drought-treated groups, respectively. Significant *P* values (*P* < 0.05) are boldfaced. Full species names can be found in Table 1.

**Supplementary Table 7** Significance tests of rewetting effects on rhizosphere soil enzyme activity per species.

| **Soil enzyme activity** | **Species** | **Ambient** | **Drought** | ***P*** |
| --- | --- | --- | --- | --- |
| CBH | QUPH | 2.88(0.40) | 2.19(0.36) | 0.455 |
|  | PHSE | 3.14(0.69) | 3.06(0.33) | 0.925 |
|  | SYBU | 2.37(0.43) | 2.72(0.61) | 0.639 |
|  | LIGL | - | 2.00(0.79) | - |
|  | ILPU | 3.51(0.25) | 2.14(0.27) | 0.131 |
|  | ORXY | 3.19(0.86) | 2.18(0.46) | 0.333 |
|  | DIKA | 7.09(1.03) | 5.68(1.09) | 0.118 |
|  | RHCH | 3.39(0.18) | 2.92(1.01) | 0.599 |
|  | TOSY | 2.40(0.55) | 2.95(0.54) | 0.518 |
|  | CAAC | 1.59(0.30) | 1.24(0.56) | 0.696 |
| BG | QUPH | 5.56(0.15) | 5.33(0.18) | 0.420 |
|  | PHSE | 5.61(0.08) | 5.66(0.05) | 0.853 |
|  | SYBU | 5.28(0.20) | 5.43(0.26) | 0.564 |
|  | LIGL | - | 5.20(0.31) | - |
|  | ILPU | 5.66(0.06) | 5.35(0.06) | 0.248 |
|  | ORXY | 5.42(0.22) | 4.82(0.33) | 0.051 |
|  | DIKA | 6.18(0.08) | 6.10(0.15) | 0.770 |
|  | RHCH | 5.71(0.04) | 5.42(0.32) | 0.279 |
|  | TOSY | 5.46(0.18) | 5.55(0.15) | 0.695 |
|  | CAAC | 4.99(0.19) | 4.76(0.32) | 0.385 |
| AP | QUPH | 6.22(0.21) | 6.32(0.11) | 0.861 |
|  | PHSE | 6.72(0.13) | 6.99(0.12) | 0.255 |
|  | SYBU | 6.44(0.20) | 6.57(0.01) | 0.507 |
|  | LIGL | - | 6.26(0.07) | - |
|  | ILPU | 6.31(0.09) | 6.29(0.18) | 0.941 |
|  | ORXY | 6.22(0.18) | 6.03(0.32) | 0.414 |
|  | DIKA | 6.75(0.11) | 6.63(0.16) | 0.616 |
|  | RHCH | 6.29(0.10) | 6.32(0.14) | 0.918 |
|  | TOSY | 6.21(0.03) | 6.38(0.17) | 0.449 |
|  | CAAC | 6.04(0.21) | 6.00(0.32) | 0.862 |
| NAG | QUPH | 4.54(0.47) | 5.25(0.08) | 0.106 |
|  | PHSE | 5.81(0.12) | 5.85(0.07) | 0.924 |
|  | SYBU | 4.00(0.15) | 4.35(0.00) | 0.464 |
|  | LIGL | - | 4.51(0.27) | - |
|  | ILPU | 4.87(0.35) | 4.62(0.37) | 0.527 |
|  | ORXY | 4.84(0.41) | 3.98(0.65) | 0.057 |
|  | DIKA | 5.47(0.31) | 5.17(0.24) | 0.458 |
|  | RHCH | 4.41(0.20) | 4.19(0.36) | 0.585 |
|  | TOSY | 4.29(0.18) | 4.48(0.30) | 0.676 |
|  | CAAC | 4.82(0.04) | 4.65(0.25) | 0.666 |

Note: Full names of the soil enzyme activities can be found in supplementary table 1. Ambient and Drought indicate mean enzyme activities (± standard error) for ambient and drought-treated groups, respectively. Significant *P* values (*P* < 0.05) are boldfaced, "-" denotes data not available. Full species names can be found in Table 1.

**Supplementary Table 8** Significance tests of rewetting effects on rhizosphere soil carbon and nutrient availability per species.

| **Soil carbon and nutrient availability** | **Species** | **Ambient** | **Drought** | ***P*** |
| --- | --- | --- | --- | --- |
| DOC | QUPH | 5.00(0.09) | 5.48(0.28) | 0.054 |
|  | PHSE | 5.34(0.15) | 5.38(0.34) | 0.850 |
|  | SYBU | 5.21(0.10) | 5.01(0.19) | 0.360 |
|  | LIGL | - | 4.96(0.14) | - |
|  | ILPU | 5.27(0.18) | 5.36(0.21) | 0.670 |
|  | ORXY | 5.13(0.14) | 5.12(0.21) | 0.851 |
|  | DIKA | 5.08(0.12) | 5.00(0.23) | 0.719 |
|  | RHCH | 4.93(0.15) | 5.14(0.05) | 0.351 |
|  | TOSY | 5.08(0.10) | 5.31(0.21) | 0.516 |
|  | CAAC | 4.91(0.13) | 4.94(0.11) | 0.902 |
| PO_4_^3-^ | QUPH | 0.93(0.35) | 1.10(0.09) | 0.676 |
|  | PHSE | 0.74(0.32) | 1.22(0.28) | 0.137 |
|  | SYBU | 1.40(0.04) | 1.35(0.18) | 0.979 |
|  | LIGL | - | 0.85(0.53) | - |
|  | ILPU | 0.50(0.19) | 1.42(0.36) | **0.005** |
|  | ORXY | 1.48(0.11) | 1.18(0.09) | 0.414 |
|  | DIKA | 2.03(0.02) | 1.96(0.22) | 0.847 |
|  | RHCH | 0.39(0.17) | 0.79(0.08) | 0.203 |
|  | TOSY | 0.83(0.00) | 1.40(0.15) | 0.112 |
|  | CAAC | 0.67(0.10) | 1.05(0.33) | 0.227 |
| NH_4_^+^ | QUPH | 2.57(0.01) | 2.53(0.06) | 0.571 |
|  | PHSE | 2.46(0.02) | 2.58(0.09) | 0.096 |
|  | SYBU | 2.46(0.05) | 2.40(0.04) | 0.967 |
|  | LIGL | - | 2.45(0.03) | - |
|  | ILPU | 2.48(0.09) | 2.65(0.06) | **0.023** |
|  | ORXY | 2.38(0.03) | 2.31(0.02) | 0.730 |
|  | DIKA | 2.58(0.12) | 2.54(0.04) | 0.642 |
|  | RHCH | 2.51(0.07) | 2.50(0.05) | 0.965 |
|  | TOSY | 2.40(0.05) | 2.46(0.06) | 0.216 |
|  | CAAC | 2.40(0.05) | 2.44(0.07) | 0.631 |
| NO_3_^-^ | QUPH | 1.30(0.06) | 1.36(0.05) | 0.326 |
|  | PHSE | 1.24(0.03) | 1.40(0.05) | **0.008** |
|  | SYBU | 1.33(0.05) | 1.32(0.06) | 0.952 |
|  | LIGL | - | 1.24(0.02) | - |
|  | ILPU | 1.33(0.04) | 1.45(0.09) | **0.043** |
|  | ORXY | 1.26(0.04) | 1.27(0.01) | 0.685 |
|  | DIKA | 1.39(0.05) | 1.32(0.04) | 0.218 |
|  | RHCH | 1.34(0.05) | 1.31(0.03) | 0.634 |
|  | TOSY | 1.31(0.05) | 1.30(0.02) | 0.996 |
|  | CAAC | 1.27(0.02) | 1.28(0.02) | 0.967 |

Note: Full names of the soil carbon and nutrient availabilities can be found in supplementary table 1. Ambient and Drought indicate mean carbon and nutrient availabilities (± standard error) for ambient and drought-treated groups, respectively. Significant *P* values (*P* < 0.05) are boldfaced, "-" denotes data not available. Full species names can be found in Table 1.

**Supplementary Table 9** Significance tests of differences in rhizosphere soil functions of drought-treated plants between drought and recovery phases.

| **Soil function** | ***df*** | ***ddf*** | ***F*** | **Drought** | **Recovery** | ***P*** |
| --- | --- | --- | --- | --- | --- | --- |
| CBH(n=76) | 1 | 9 | 35.31 | 1.22(0.16) | 2.71(0.37) | **0.001** |
| BG(n=76) | 1 | 9 | 83.72 | 4.56(0.07) | 5.36(0.12) | **0.001** |
| AP(n=76) | 1 | 9 | 54.43 | 5.74(0.05) | 6.38(0.09) | **0.001** |
| NAG(n=76) | 1 | 9 | 32.20 | 3.99(0.12) | 4.70(0.18) | **0.001** |
| DOC(n=76) | 1 | 9 | 40.48 | 4.88(0.04) | 5.17(0.06) | **0.001** |
| PO_4_^3-^(n=76) | 1 | 9 | 13.03 | 1.54(0.07) | 1.23(0.11) | **0.006** |
| NH_4_^+^(n=76) | 1 | 9 | 1.49 | 2.46(0.03) | 2.49(0.03) | 0.254 |
| NO_3_^-^(n=76) | 1 | 9 | 1.37 | 1.18(0.12) | 1.32(0.02) | 0.272 |

Note: Full names of the soil function can be found in supplementary table 1. Data for ambient treatment of tree species *Lithocarpus glaber* (LIGL) during recovery phase are missing due to insufficient plant samples. *df*, numerator degrees of freedom; *ddf*, denominator degrees of freedom (these reflect residual degrees of freedom for calculating sampling phase effects of drought-treated groups across 10 species). *F* and *P* indicate *F* ratios and the *P* values of the significance tests, respectively. Numbers in parentheses after soil functions indicate sample sizes. Drought and Recovery show mean values (± standard error) for drought-treated groups during drought and recovery phases, respectively. Significant *P* values (*P* < 0.05) are highlighted in boldface.

**Supplementary Table 10** Differences in the drought and rewetting effects on rhizosphere soil functions between habitat-generalist and -specialist species.

| **Treatment** | **Soil function** | **df** | **ddf** | **F** | **Generalist** | **Specialist** | ***P*** |
| --- | --- | --- | --- | --- | --- | --- | --- |
| Drought | CBH(n=79) | 1 | 8 | 2.41 | -3.46(0.43) | -2.34(0.35) | 0.16 |
|  | BG(n=79) | 1 | 8 | 0.77 | -1.18(0.18) | -1.33(0.15) | 0.41 |
|  | AP(n=79) | 1 | 8 | 0.94 | -0.71(0.13) | -0.84(0.10) | 0.36 |
|  | NAG(n=79) | 1 | 8 | 0.31 | -1.34(0.21) | -1.47(0.18) | 0.59 |
|  | DOC(n=79) | 1 | 8 | 0.15 | -0.22(0.12) | -0.28(0.10) | 0.71 |
|  | PO_4_^3-^(n=79) | 1 | 8 | 0.02 | -0.04(0.08) | -0.02(0.07) | 0.89 |
|  | NH_4_^+^(n=79) | 1 | 8 | 2.25 | -0.03(0.06) | -0.15(0.05) | 0.17 |
|  | NO_3_^-^(n=79) | 1 | 8 | 1.09 | 0.25(0.15) | 0.05(0.12) | 0.33 |
| Rewetting | CBH(n=71) | 1 | 7 | 1.37 | -0.97(0.52) | -0.35(0.37) | 0.28 |
|  | BG(n=71) | 1 | 7 | 0.35 | -0.12(0.16) | -0.20(0.11) | 0.57 |
|  | AP(n=71) | 1 | 7 | 0.19 | -0.03(0.14) | 0.05(0.10) | 0.67 |
|  | NAG(n=71) | 1 | 7 | 0.03 | -0.11(0.24) | -0.06(0.17) | 0.87 |
|  | DOC(n=71) | 1 | 7 | 2.45 | -0.07(0.12) | 0.15(0.09) | 0.16 |
|  | PO_4_^3-^(n=71) | 1 | 7 | 0.01 | 0.26(0.20) | 0.29(0.14) | 0.91 |
|  | NH_4_^+^(n=71) | 1 | 7 | 0.10 | 0.04(0.04) | 0.03(0.03) | 0.77 |
|  | NO_3_^-^(n=71) | 1 | 7 | 0.12 | 0.02(0.04) | 0.04(0.03) | 0.74 |

Note: Full names of the soil function can be found in supplementary table 1. *df*, numerator degrees of freedom; *ddf*, denominator degrees of freedom (these reflect residual degrees of freedom for drought and recovery treatment effects calculations across 10 species). *F* and *P* indicate *F* ratios and the *P* values of the significance tests, respectively. Numbers in parentheses after soil functions indicate sample sizes. Generalist and Specialist show mean treatment effects (± standard error) of drought and recovery for habitat-generalist and -specialist species, respectively. Significant *P* values (*P* < 0.05) are highlighted in boldface.

**Supplementary Table 11** Differences in the effects of drought and rewetting effects on rhizosphere soil functions between hilltop-specific and valley-specific species.

| **Treatment** | **Soil function** | **df** | **ddf** | **F** | **Hilltop** | **Valley** | ***P*** |
| --- | --- | --- | --- | --- | --- | --- | --- |
| Drought | CBH(n=47) | 1 | 4 | 0.08 | -2.49(0.58) | -2.27(0.42) | 0.79 |
|  | BG(n=47) | 1 | 4 | 0.11 | -1.28(0.26) | -1.35(0.19) | 0.76 |
|  | AP(n=47) | 1 | 4 | 1.24 | -0.91(0.19) | -0.79(0.14) | 0.33 |
|  | NAG(n=47) | 1 | 4 | 0.72 | -1.34(0.29) | -1.52(0.21) | 0.44 |
|  | DOC(n=47) | 1 | 4 | 0.86 | -0.13(0.17) | -0.35(0.12) | 0.41 |
|  | PO_4_^3-^(n=47) | 1 | 4 | 9.58 | -0.23(0.11) | 0.10(0.08) | **0.04** |
|  | NH_4_^+^(n=47) | 1 | 4 | 0.29 | -0.11(0.08) | -0.17(0.06) | 0.62 |
|  | NO_3_^-^(n=47) | 1 | 4 | 1.09 | 0.21(0.13) | -0.03(0.09) | 0.36 |
| Rewetting | CBH(n=44) | 1 | 4 | 0.00 | -0.35(0.58) | -0.32(0.42) | 0.99 |
|  | BG(n=44) | 1 | 4 | 0.84 | -0.08(0.21) | -0.25(0.15) | 0.41 |
|  | AP(n=44) | 1 | 4 | 1.95 | 0.16(0.18) | -0.01(0.13) | 0.24 |
|  | NAG(n=44) | 1 | 4 | 3.24 | 0.35(0.28) | -0.29(0.21) | 0.15 |
|  | DOC(n=44) | 1 | 4 | 1.01 | 0.23(0.16) | 0.10(0.12) | 0.37 |
|  | PO_4_^3-^(n=44) | 1 | 4 | 0.07 | 0.33(0.22) | 0.25(0.16) | 0.81 |
|  | NH_4_^+^(n=44) | 1 | 4 | 0.23 | 0.05(0.05) | 0.02(0.04) | 0.66 |
|  | NO_3_^-^(n=44) | 1 | 4 | 11.78 | 0.12(0.03) | 0.00(0.02) | **0.03** |

Note: Full names of the soil function can be found in supplementary table 1. *df*, numerator degrees of freedom; *ddf*, denominator degrees of freedom (these reflect residual degrees of freedom for drought and recovery treatment effects calculations across 10 species). *F* and *P* indicate *F* ratios and the *P* values of the significance tests, respectively. Numbers in parentheses after soil functions indicate sample sizes. Hilltop and Valley show mean treatment effects (± standard error) of drought and recovery for hilltop-specific and valley-specific species, respectively. Significant *P* values (*P* < 0.05) are highlighted in boldface.

**Supplementary methods**

**Amplicon sequencing of root endosphere fungi and root mycorrhizal fungi fraction**

We collected one sample of fine root (< 2 mm diameter) for one plant per species per block to conduct amplicon sequencing of root endosphere fungi (Edwards et al., 2018). We used flame-sterilized scissors to cut ~5 cm of root immediately below the root-shoot junction. We placed the tissue in a sterile 50 mL tube with 20 mLof autoclaved Phosphate Buffered Saline (PBS) solution. Endosphere samples were collected by washing the roots in fresh PBS to further discard any remaining soil and sonicating them four times (50 to 60Hz for 30 s) to ensure that all microbes were removed from the root surface. The sonicated roots constituted the endosphere compartment. We stored the roots at -80 °C until DNA extraction in the same day.

The root endosphere fraction was pre-homogenized before the DNA extraction by bead beating for 1 minute (Mini Beadbeater, Biospec Products). Total genome DNA from samples was extracted using CTAB/SDS method. DNA concentration and purity were monitored on 1% agarose gels. According to the concentration, DNA was diluted to 1 ug/μL using sterile water. For root endosphere fungi, the ITS1-1F region was amplified using the universal primers ITS1F (CTTGGTCATTTAGAGGAAGTAA) and ITS2 (GCTGCGTTCTTCATCGATGC) (Johnston-Monje et al., 2021). No measures were taken to minimize amplification of host chloroplast and mitochondria sequences. All PCR reactions were carried out with 15μL of Phusion® High-Fidelity PCR Master Mix (New England Biolabs); 0.2 μM of forward and reverse primers, and about 10 ng template DNA. Thermal cycling consisted of initial denaturation at 98℃ for 1 min, followed by 30 cycles of denaturation at 98℃ for 10 s, annealing at 50 ℃ for 30 s, and elongation at 72 ℃for 30 s and 72 ℃ for 5 min. Mix same volume of IX loading buffer (contained SYB green) with PCR products and operate electrophoresis on 2% agarose gel for detection. PCR products was mixed in equidensity ratios. Then, mixture PCR products was purified with Qiagen Gel Extraction Kit (Qiagen, Germany). Sequencing libraries were generated using TruSeq® DNA PCR-Free Sample Preparation Kit (Illumina, USA) following manufacturer's recommendations and index codes were added. The library quality was assessed on the Qubit@2.0 Fluorometer (Thermo Scientific) and Agilent Bioanalyzer 2100 system. At last, the library was sequenced on an Illumina NovaSeq platform and 250 bp paired-end reads were generated.

Paired-end reads were assigned to samples based on their unique barcode and truncated by cutting off the barcode and primer sequence. Paired-end reads were merged using FLASH (V1.2.1 1) (Magoc and Salzberg, 2011). Quality filtering on the raw tags were performed using the FASTP software to obtain high-quality Clean Tags (Bokulich et al., 2013). The tags were compared with the reference database using UCHIME algorithm to detect chimera sequences, and then the chimera sequences were removed (Edgar et al., 2011). For the Effective Tags obtained previously, denoise was performed with DADA2 to obtain initial ASVs. Non-fungal ASVs (chloroplasts) were removed. We performed rarefaction normalization using the ‘*rrarefy*’function in the vegan package (vegan Development Team, 2023) to standardize sequencing depth across fungal samples, establishing the baseline dataset for subsequent analyses. We identified functional guilds to root endosphere fungal ASVs using the FUNGuildR package (FungalTraits Team, 2023) with the UNITE database (Nguyen et al., 2016), based on taxonomic annotations of individual clusters. Mycorrhizal fungal proportion (MF) was calculated as the relative abundance ratio of arbuscular mycorrhizal fungi [AMF] and ectomycorrhizal fungi [EMF]) to all fungi.

**References**

Bokulich, N. A., S. Subramanian, J. J. Faith, D. Gevers, J. I. Gordon, R. Knight, D. A. Mills & J. G. Caporaso 2013. Quality-filtering vastly improves diversity estimates from Illumina amplicon sequencing. *Nature Methods,* 10, 1**,** 57-59.

Edgar, R. C., B. J. Haas, J. C. Clemente, C. Quince & R. Knight 2011. UCHIME improves sensitivity and speed of chimera detection. *Bioinformatics,* 27, 16**,** 2194-2200.

Edwards, J., C. Santos-Medellín & V. Sundaresan 2018. Extraction and 16S rRNA Sequence Analysis of Microbiomes Associated with Rice Roots. *Bio-Protocol,* 8, 12.

FungalTraits Team 2023. FUNGuildR: Fungal functional guild annotation in R GitHub.

Johnston-Monje, D., J. P. Gutierrez & L. A. B. Lopez-Lavalle 2021. Seed-Transmitted Bacteria and Fungi Dominate Juvenile Plant Microbiomes. *Frontiers in Microbiology,* 12**,** 1-25.

Magoc, T. & S. L. Salzberg 2011. FLASH: fast length adjustment of short reads to improve genome assemblies. *Bioinformatics,* 27, 21**,** 2957-2963.

Nguyen, N. H., Z. W. Song, S. T. Bates, S. Branco, L. Tedersoo, J. Menke, J. S. Schilling & P. G. Kennedy 2016. FUNGuild: An open annotation tool for parsing fungal community datasets by ecological guild. *Fungal Ecology,* 20**,** 241-248.

vegan Development Team 2023. vegan: Community Ecology Package. R package version 2.6-4 ed. CRAN.
